# A MAFG-lncRNA axis links systemic nutrient abundance to hepatic glucose metabolism

**DOI:** 10.1038/s41467-020-14323-y

**Published:** 2020-01-31

**Authors:** Marta Pradas-Juni, Nils R. Hansmeier, Jenny C. Link, Elena Schmidt, Bjørk Ditlev Larsen, Paul Klemm, Nicola Meola, Hande Topel, Rute Loureiro, Ines Dhaouadi, Christoph A. Kiefer, Robin Schwarzer, Sajjad Khani, Matteo Oliverio, Motoharu Awazawa, Peter Frommolt, Joerg Heeren, Ludger Scheja, Markus Heine, Christoph Dieterich, Hildegard Büning, Ling Yang, Haiming Cao, Dario F. De Jesus, Rohit N. Kulkarni, Branko Zevnik, Simon E. Tröder, Uwe Knippschild, Peter A. Edwards, Richard G. Lee, Masayuki Yamamoto, Igor Ulitsky, Eduardo Fernandez-Rebollo, Thomas Q. de Aguiar Vallim, Jan-Wilhelm Kornfeld

**Affiliations:** 1 Functional Genomics and Metabolism Unit, Department for Biochemistry and Molecular Biology, University of Southern Denmark, Campusvej 55, 5230 Odense M, Denmark; 2 Max Planck Institute for Metabolism Research, Gleueler Strasse 50, 50931 Cologne, Germany; 3 Cologne Cluster of Excellence—Cellular Stress Responses in Ageing-associated Diseases (CECAD), Medical Faculty, University of Cologne, Joseph-Stelzmann-Str. 26, 50931 Cologne, Germany; 4 Department of Biological Chemistry, University of California, Los Angeles (UCLA), 650 Charles E. Young Drive South, Los Angeles, CA 90095 USA; 5 Department of Medicine, Division of Cardiology, UCLA, 650 Charles E. Young Drive South, Los Angeles, CA 90095 USA; 6 Izmir Biomedicine and Genome Center (IBG), Mithatpasa Ave. 58/5, 35340 Izmir, Turkey; 7 Department of Medical Biology and Genetics, Graduate School of Health Sciences, Dokuz Eylul University, Mithatpasa Ave. 1606, 35330 Izmir, Turkey; 8 Diabetes Research Center, Research Institute, National Center for Global Health and Medicine, Tokyo, 162-8655 Japan; 9 Department of Biochemistry and Molecular Cell Biology, Martinistraße 52, 20246 Hamburg, Germany; 10 Section of Bioinformatics and Systems Cardiology, Klaus Tschira Institute for Integrative Computational Cardiology, University Hospital Heidelberg, Im Neuenheimer Feld 669, 69120 Heidelberg, Germany; 11 Institute of Experimental Hematology, Hanover Medical School, Carl-Neuberg-Str. 1, 30625 Hannover, Germany; 12 Cardiovascular Branch, National Heart Lung and Blood Institute, Bethesda, MD 20892 USA; 13 Islet Cell and Regenerative Biology, Joslin Diabetes Center, Department of Medicine, Brigham and Women’s Hospital, Harvard Stem Cell Institute, Harvard Medical School, Boston, 02215 MA USA; 14 CECAD in vivo Research Facility, Medical Faculty, University of Cologne, Joseph-Stelzmann-Str. 26, 50931 Cologne, Germany; 15 Department of General and Visceral Surgery, University Hospital Ulm, Albert-Einstein Allee 93, 89081 Ulm, Germany; 16 IONIS Pharmaceuticals, Carlsbad, CA 92010 USA; 17 Department of Medical Biochemistry, Tohoku Medical Megabank Organization, Sendai, 980-8573 Japan; 18 Department of Biological Regulation, Weizmann Institute of Science, Rehovot, 76100 Israel; 19 Present Address: Lewis Katz School of Medicine, Temple University, Philadelphia, PA 19140 USA

**Keywords:** CRISPR-Cas9 genome editing, Long non-coding RNAs, Transcriptomics, Obesity

## Abstract

Obesity and type 2 diabetes mellitus are global emergencies and long noncoding RNAs (lncRNAs) are regulatory transcripts with elusive functions in metabolism. Here we show that a high fraction of lncRNAs, but not protein-coding mRNAs, are repressed during diet-induced obesity (DIO) and refeeding, whilst nutrient deprivation induced lncRNAs in mouse liver. Similarly, lncRNAs are lost in diabetic humans. LncRNA promoter analyses, global cistrome and gain-of-function analyses confirm that increased MAFG signaling during DIO curbs lncRNA expression. Silencing *Mafg* in mouse hepatocytes and obese mice elicits a fasting-like gene expression profile, improves glucose metabolism, de-represses lncRNAs and impairs mammalian target of rapamycin (mTOR) activation. We find that obesity-repressed *LincIRS2* is controlled by MAFG and observe that genetic and RNAi-mediated *LincIRS2* loss causes elevated blood glucose, insulin resistance and aberrant glucose output in lean mice. Taken together, we identify a MAFG-lncRNA axis controlling hepatic glucose metabolism in health and metabolic disease.

## Abstract (web summary)

Despite widespread transcription of LncRNA in mammalian systems, their contribution to metabolic homeostasis at the cellular and tissue level remains elusive. Here Pradas-Juni et al. describe a transcription factor–LncRNA pathway that couples hepatocyte nutrient sensing to regulation of glucose metabolism in mice.

## Introduction

Cellular and organism-level energy homeostasis and nutrient partitioning are instrumental for survival. In higher organisms, multi-organ systems evolved to react to shifts in energy supply by storing (anabolic) or metabolizing (catabolic) nutrients, for instance by conversion of simple sugars to storage macromolecules like glycogen or by glucose catabolism, according to caloric demands^1^. A key process for mounting appropriate responses to altered nutrient availabilities are abundance, localization, and nutrient-induced activation of transcriptional networks that couple energy states to appropriate changes in gene expression^2^. These ancient molecular circuits ensured survival of animals during (historically frequent) food shortages. In contrast, the exposure to constant calorie overload, coupled with sedentary lifestyles, results in excess storage of nutrients, and concomitantly, obesity and obesity-associated maladies. Globally, two billion individuals are considered overweight or obese and exhibit elevated risks of developing severe comorbidities such as cardiovascular disease^3^, artherosclerosis^4^, type 2 diabetes mellitus (T2D), liver steatosis, or nonalcoholic fatty liver disease (NAFLD) and nonalcoholic steatohepatitis (NASH)^5^. Therefore, understanding how nutrient-sensitive signaling networks are controlled during conditions of energy surplus, and whether this can be prevented in obesity, will be instrumental in designing effective therapies in the future.

Remarkable for living in seemingly postgenomic times, we currently witness a paradigm shift in understanding our genomes and the information contained therein: multinational sequencing efforts like ENCODE^6^, FANTOM^7^, or NONCODE^8^, together with increasing RNA-Sequencing (RNA-Seq) capabilities and reduced costs, led to the intriguing discovery that, whereas only 1–2% of genomic information encodes protein-coding mRNAs, more than 70% of DNA is transcribed across developmental space and time^9,10^. This led to the identification of thousands of microRNAs, and more recently, long noncoding RNAs (lncRNAs)^11,12^ in mice and humans^13^. The identification of these many lncRNAs in silico and ascribing a specific function to each has been challenging^14^. Functions identified to date include microRNA scavenging^15^, chromatin sequestration^16^, 3D genome organization^17^, chromatin modifier recruitment^18^, lncRNA–DNA triplex formation^19^, and small-molecule protein complex formation^20^ to control mRNA translation^15^ and RNA–RNA crosstalk^21^.

Investigators have only begun to ascribe specific in vivo functions for lncRNAs in cellular energy homeostasis and/or metaboregulatory signaling circuits. Such functions of lncRNAs include control of hepatic cholesterol biosynthesis^22^, de novo lipogenesis and systemic lipid homeostasis^23,24^, beta-oxidation^25^, glucose homeostasis^26,27^, and regulation of insulin sensitivity in the liver^27^. Understanding the molecular details of these regulatory processes may lead to innovative approaches where repression of specific lncRNAs may prove useful in the treatment of metabolic diseases.

By performing RNA-Seq in mouse models for dietary and genetic obesity, T2D, and livers from diabetic human patients, we find that nutrient excess and refeeding (RF) favors lncRNA repression, whereas fasting induces lncRNA expression. Intriguingly, protein-coding mRNAs are not affected accordingly. In silico analyses of lncRNA and mRNA promoters, and integration of MAFG ChIP-Seq with MAFG gain-of-function RNA-Seq data confirm that elevated MAFG signaling in obesity and RF is transcriptionally linked to lncRNA repression. In vitro and in vivo loss of MAFG in hepatocytes controls glucose production, improves glucose metabolism during obesity, and induces lncRNAs, whereas MAFG gain of function represses hepatic lncRNAs. Intriguingly, MAFG loss prevents insulin-evoked activation of mTORC1 signaling, thus presumably interfering with protein translation in hepatocytes. We further observe that obesity-repressed *LincIRS2* is negatively controlled by MAFG and CRISPR–Cas9-mediated knockout, or antisense-mediated RNA interference of *LincIRS2* causes hyperglycemia, insulin resistance, likely caused by alterations in glucogenic gene expression in lean mice.

## Results

### Nutrient states elicit opposing effects on mRNA and lncRNAs

**Fig. 1:**
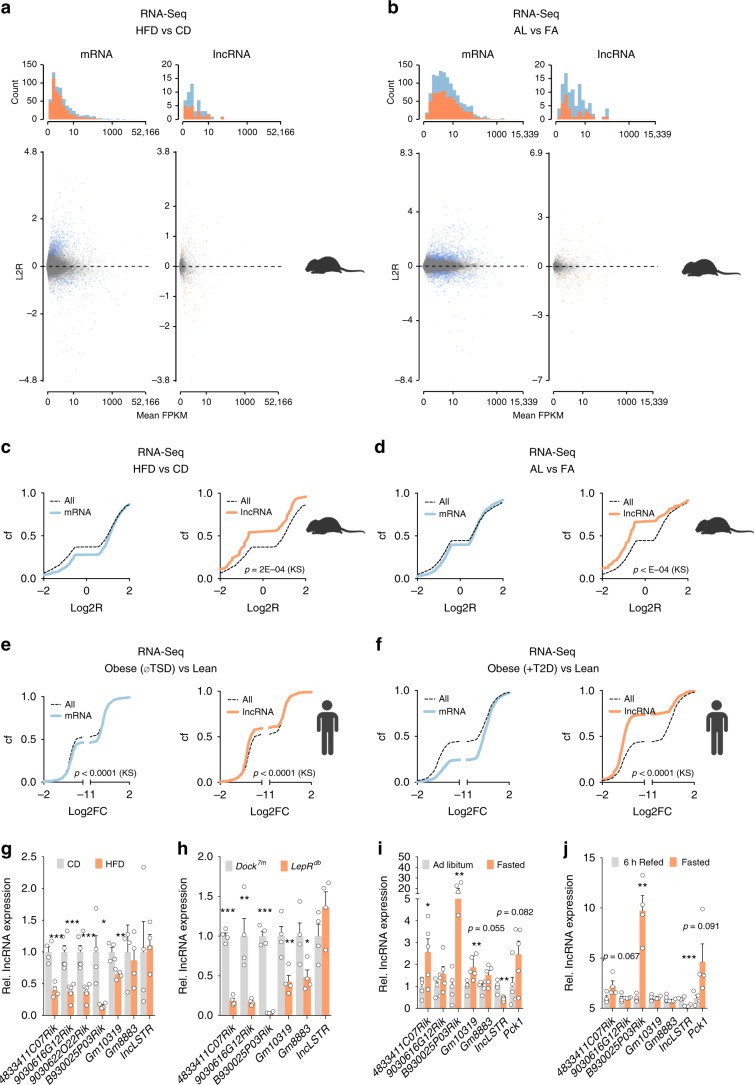
Systemic nutrient states elicit opposing effects on liver mRNA and lncRNA expression. **a**, **b** Histogram plot of read counts (*top*), scatter plot of reads counts vs. log2-transformed expression ratios (Log2R, *bottom*) and **c**, **d** cumulative frequency distribution (Cf) of Log2R of hepatic protein-coding mRNA (blue) and lncRNA (orange) expression changes. Data are from total RNA-Sequencing (RNA-Seq) in the liver of C57BL/6N mice after **a**, **c** HFD vs. (vs) CD feeding (*n* = 3 each) or **b**, **d** ad libitum feeding (AL) vs. 16 h of fasting (FA) (*n* = 4 each). **e** Log2R Cf of mRNA (blue) and lncRNA (orange) expression changes in the liver from lean (L) vs. obese (OB) patients without T2D (*n* = 4 per group). **f** Log2R Cf of mRNA (blue) and lncRNA (orange) expression changes in the liver of lean (L) vs. obese patients with T2D (*n* = 4 per group). **g**–**j** Quantitative reverse-transcription (RT) polymerase chain reaction (qPCR) for L-DIO-lncRNA expression in mice exposed to **g** HFD vs. CD feeding (*n* = 4–6). **h** *Lepr*^*db*^ vs. *Dock*^7*m*^ (*n* = 4). **i** AL vs. FA (*n* = 5 each) and **j** RF vs. FA (*n* = 4 each). **c**–**f** Statistical differences between mRNAs and lncRNAs were assessed using nonparametric Kolgomorov–Smirnov (KS) tests. *p* Values are given in the panels. Bar graphs represent mean ± s.e.m. with all data points plotted and differences in (**g**–**j**) were calculated using unpaired, two-tailed Student’s *t* tests. *p < 0.05, **p < 0.01, ***p < 0.001. Source data are provided as a Source Data file. Icons in **a**–**f** were created with BioRender.com.

To identify lncRNAs that are implicated in the development of liver disease pathologies in diet-induced obesity (DIO), for instance insulin resistance, steatosis, and liver inflammation, 6-week-old C57BL/6N mice were fed a high-fat diet (HFD) or control diet (CD). After 30 weeks, hepatic RNA was isolated and total RNA-Sequencing (RNA-Seq) performed. This approach identified 583 mRNAs and 50 lncRNAs that were significantly (*p* value (pV) < 0.05 by Student’s *t* test, false-discovery rate < 0.05, and CuffDiff function significant = “yes” for Benjamini–Hochberg correction for multiple testing) altered after HFD feeding (Fig. 1a, Supplementary Data 1). Performing Ingenuity Pathway Analysis (IPA) confirmed activation of transcription factor (TF) networks and signaling pathways known to be induced in the liver under anabolic/nutrient-rich conditions. These included insulin receptor (IR), Forkhead Box O1 (FOXO1), and Sterol Regulatory Element Binding Transcription Factor 1C (SREBP1C) pathways (Supplementary Fig. 1a–c). We interpreted these transcriptional changes as a reflection of chronic nutrient exposure that in turn triggers anabolic TF pathways in the liver.

When performing differential gene expression (DGE) analysis independently for protein-coding or lncRNA transcripts (gene biotypes from Ensembl Biomart^28^), we observed a significant cumulative downregulation of lncRNAs as compared with protein-coding mRNAs (Fig. 1c) in obese livers using nonparametric Kolmogorov–Smirnov tests. Prompted by this difference in mRNA vs. lncRNA regulation after HFD feeding, we repeated the analysis after physiological nutrient changes outside of obesity and metabolic disease. Comparing transcriptomes from ad libitum (AL)-fed C57BL/6N (wild-type) mice compared with mice fasted for 16 h (FA, Fig. 1b, Supplementary Data 2), we observed 1165 mRNAs and 92 lncRNAs differentially regulated. In contrast to lncRNA repression in obesity, lncRNAs were induced after fasting when compared with mRNAs (Fig. 1d). Short-term (6 h) RF altered 587 mRNAs and 59 lncRNAs (Supplementary Data 3) with similar trends for induction of lncRNAs as compared with mRNAs (Supplementary Fig. 1d). We performed RNA-Seq in *BKS.Cg-Dock*^*7m+/+*^; *LepR*^*db*^*/J* (termed *LepR*^*db*^) mice, a genetic model of obesity and T2D, and observed 959 mRNAs and 83 lncRNAs significantly changed (Supplementary Data 4). Again, hepatic lncRNAs were decreased compared with mRNAs (Supplementary Fig. 1e) when we compared *LepR*^*db*^ vs. genetic background-matched *misty* (*Dock*^7*m*^) mice, although to a lesser degree than in DIO. To determine whether similar patterns are observed in human subjects, we analyzed RNA-Seq data from liver biopsies from a cohort of lean, obese (without T2D), and obese, T2D patients (*n* = 4 per group)^29^. Consistent with our mouse studies, obesity and T2D in humans were associated with repressions in lncRNAs vs. mRNAs (Fig. 1e, f and Supplementary Data 5).

To determine if our metabolically regulated lncRNAs represent liver-enriched transcripts, we performed RNA-Seq in seven tissues of lean C57BL/6N mice and identified clusters of lncRNAs enriched in each tissue (Supplementary Fig. 1f). Within the liver-enriched cluster, seven DIO-associated lncRNAs (termed L-DIO-lncRNAs), including *4833411C07Rik*, *9030616G12Rik*, *9030622O22Rik*, *B930025P03Rik*, *Gm10319*, *Gm8883*, and previously reported *lncLSTR*^24^ (Supplementary Fig. 1g) matched our criteria: the expression of L-DIO-lncRNA expression was confined to hepatocytes compared with non-parenchymal liver cell types (Supplementary Fig. 1h). With the exception of *Gm8883*, L-DIO-lncRNAs were located in the nucleus (Supplementary Fig. 1i), and with exception of *Gm10319*, exhibited low protein-coding potential (using two independent algorithms, CPAT^30^ and CPC^14^, Supplementary Fig. 1j, k). Using quantitative polymerase chain reaction (qPCR) we confirmed L-DIO-lncRNAs to be reduced by HFD vs. CD feeding (Fig. 1g), in *LepR*^*db*^ vs. *Dock*^7*m*^ (Fig. 1h), in AL vs. FA (Fig. 1i), and in RF vs. FA mice (Fig. 1j). Thus, our extensive RNA-Seq analyses and qPCR validation identified an inverse correlation of nutrient levels with lncRNAs across several mouse models of altered energy homeostasis and metabolically compromised humans and identified metabolically regulated (liver-selective) lncRNAs.

### Liver MAFG links high nutrient states to lncRNA repression

**Fig. 2:**
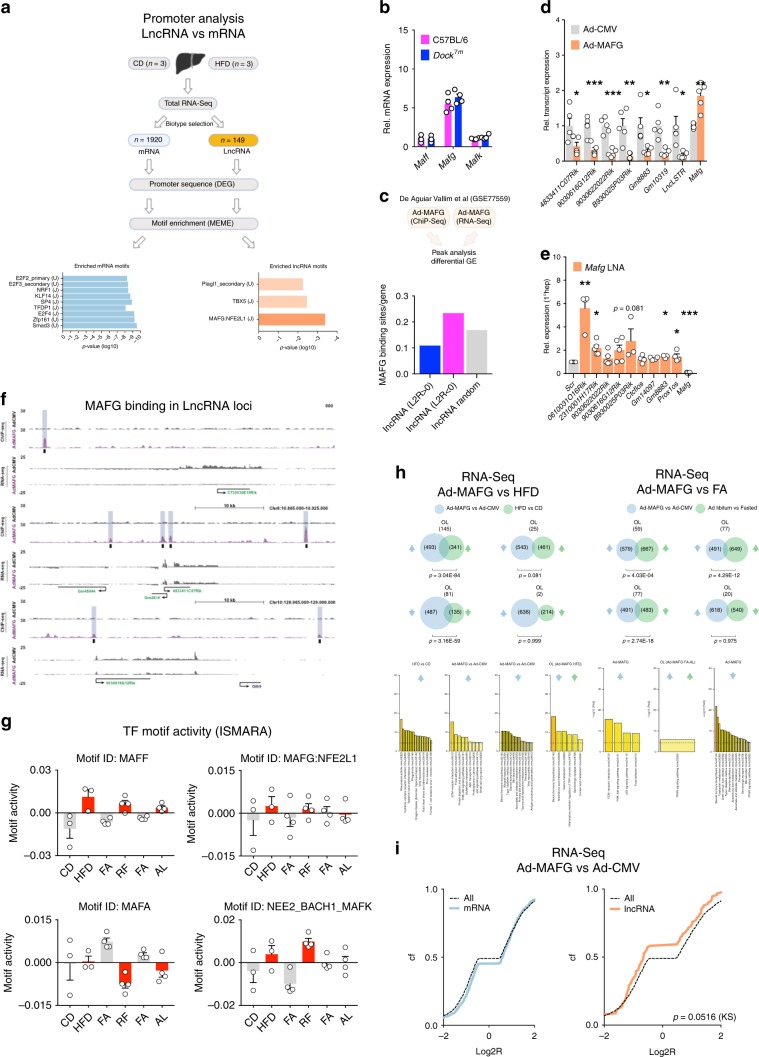
Obesity-associated increase in MAFG signaling links nutrient states to lncRNA repression. **a** Illustration of transcription factor-binding site (TFBS) analysis and a list of TFBS enriched in promoters of mRNAs (blue) or lncRNAs (orange) affected by HFD vs. CD feeding (*n* = 3 each). TFBS is from UniProbe (U) or Jaspar (J). **b** qPCR of smMAF (*Maff*, *Mafg*, and *Mafk*) expression in C57BL/6N and *Dock*^7*m*^ mice (*n* = 4 each). **c** Published BLRP ChIP-seq in livers of mice transduced with adenovirus (Ad)-overexpressing BLRP-MAFG fusion proteins (GEO ID: GSE77559)^40^ was used as a proxy for MAFG cistromes in the liver. BLRP-MAFG peaks per gene were determined in a ±50-kb window around the TSS (defined as ±1 bp from gene start) of expressed, induced, or repressed lncRNAs by HFD using window function in bedtools^75^. **d** qPCR of L-DIO-lncRNAs and *Mafg* mRNA in livers of Ad-MAFG vs. Ad-CMV mice (*n* = 5 each). **e** qPCR of lncRNA exhibiting MAFG binding within TSS ± 50 kb, as identified by BLRP-MAFG ChIP-Seq and *Mafg* expression after transfection of primary hepatocytes with 100 nM *Mafg* or Scr LNAs, *n* = 4–5 experiments, each performed in triplicates. **f** UCSC Genome Browser with expression of selected lncRNAs (“RNA-Seq”) and BLRP-MAFG binding (“ChIP-Seq” from Vallim et al.^40^) in the liver. **g** Motif activity of smMAF and MAFA TFBS across nutritional states and HFD feeding analyzed using ISMARA^41^ (*n* = 3–4 per group). **h** Venn diagram illustrating DGE overlap and enriched gene ontology (GO) categories shared between indicated nutritional states, diets, and genotypes. **i** Log2R Cf of mRNA (blue) and lncRNA (orange) expression changes in livers of Ad-MAFG vs. Ad-CMV mice (*n* = 4 each). **d**, **e** Bar graphs represent mean ± s.e.m. with all data points plotted, and statistical differences were calculated using **d** unpaired or **e** paired, two-tailed Student’s *t* test. **h** Significance of gene overlaps was calculated by using a hypergeometric distribution: *p < 0.05, **p < 0.01, ***p < 0.001. Source data are provided as a Source Data file. Illustration in (**a**) was created with BioRender.com.

Our data suggested that many lncRNAs are discordantly affected by (patho)-physiological changes in nutrient states when compared with mRNAs. We hypothesized that these differences between mRNAs and lncRNAs could reflect differences in TF- binding site (TFBS) occurence in promoters of both gene sets. These differences in promoter architecture could, in turn, be differentially transactivated by nutrient-sensitive signaling pathways, leading to anticorrelative regulation of lncRNAs vs. mRNAs during obesity and T2D as observed. Our hypothesis built on in silico analyses of chromatin-state maps^31^ and validation studies in human cell lines^32^ that suggest preexisting promoter differences between lncRNAs and mRNAs. To identify TF pathways that control lncRNAs and mRNAs via distinct regulatory programs, we first analyzed putative promoter sequences (−800 bp to +100 bp around transcriptional start sites, TSS) from an extended set of 1920 mRNAs and 149 lncRNAs affected by HFD (*p* value < 0.1, CuffDiff DGE output). Next, we used AME^33^ (MEME suite^34^) to call differences in TF motif occurence between lncRNA and mRNA promoters. Consistent with previous reports^31,35^, we observed that CpG-rich motifs were overrepresented in mRNA promoters, particularly motifs recognized by the E2F family of TF (e.g., E2F2–E2F4). In contrast, lncRNA promoters were enriched for MAFG:NFE2L1 (*V-Maf Avian Musculoaponeurotic Fibrosarcoma Oncogene Homolog G:Nuclear Factor, Erythroid 2 Like 1*) motifs (Fig. 2a). This finding was consistent with reports demonstrating that TFBS recognized by MAFG or other members of the small MAF (smMAF) TF family (MAFF, MAFG, and MAFK) is overrepresented in lncRNA promoters^31,32,35^. In a second step and given the reported gene-repressive properties of MAFG homodimers^36,37^, we wanted to exclude that MAFG motifs occurred in lncRNA promoters simply because lncRNA tends to be more repressed than mRNAs during DIO. We thus performed de novo TF motif enrichment analyses of the putative promoter sequences (−800/+100 bp around TSS) in HOMER^38^ separately for induced and repressed genes, and confirmed that smMAF motifs were enriched in lncRNA promoters affected by DIO vs. protein-coding RNAs in both gene sets (Supplementary Fig. 2a, b). Taken together, our data suggested that MAFG, or other smMAF TFs, is enriched in lncRNA promoters as compared with mRNAs, and could elicit anticorrelative transcriptional effects, for instance repress hepatic lncRNAs while, at the same time, inducing levels of specific mRNAs.

Because smMAF TF is considered as functionally redundant^39^, we next asked which smMAF constituted the prevalent liver isoform, and presumably, exerted the strongest effect on lncRNA repression during obesity. Using RNA-Seq, we found that *Mafg* accounted for the majority of smMAF transcripts in livers from lean C57BL/6N and *Dock*^7*m*^ mice (Fig. 2b). Integrating RNA-Seq analysis from mice injected with *Mafg* cDNA-expressing adenoviruses (Ad-MAFG), and public MAFG chromatin immunoprecipitation coupled to sequencing (ChIP-Seq) datasets^40^, we found that MAFG preferentially bound lncRNAs repressed during DIO (Fig. 2c). We performed qPCR in livers from Ad-MAFG-treated mice and confirmed that the seven L-DIO-lncRNAs (Fig. 2d) were repressed after *Mafg* overexpression. Conversely, locked nucleic acid (LNA)-mediated knockdown of *Mafg* (Supplementary Fig. 2c), but not *Maff* or *Mafk* (Supplementary Fig. 2c–e), in primary hepatocytes derepressed a set of lncRNAs containing MAFG-binding sites (Fig. 2e) as determined by ChIP-Seq (Fig. 2f). These data supported the hypothesis that MAFG represses specific lncRNAs in DIO, suggesting MAFG as TF whose activity changes during metabolic states. To investigate the degree of smMAF signaling activity in lean and obese livers, we applied ISMARA^41^, a web-based tool modeling genome-wide expression changes in RNA-Seq data by predicting the underlying combination of TFBS. As proof of principle and as expected, we found an induction of inflammatory CEBPB and RELA-REL-NFKB1 gene programs in obese liver (Supplementary Fig. 2f, g). When comparing all combinations of nutrient-rich (HFD, AL, and RF) vs. nutrient-poor conditions (CD and FA) we found that anabolic conditions exhibited higher smMAF (NFE2:BACH1:MAFK, MAFG:NFE2L1, and MAFF) motif activities in the liver, while large MAFA^42^-dependent TFBS activities were reduced after DIO or RF (Fig. 2g). Importantly, RNA-Seq from HFD and Ad-MAFG-treated mice revealed a significant overlap of directional gene expression changes and overlapping gene categories (Fig. 2h, *left*), while Ad-MAFG and fasting gene sets showed anticorrelative trends (Fig. 2h, *right*). Finally, RNA-Seq in Ad-MAFG livers revealed trends toward repression of lncRNAs compared with mRNAs (Fig. 2i, Supplementary Data 6). Thus, our data suggested that acute (RF and AL) and chronic (DIO) nutrient exposure is associated with increased smMAF signaling, and that gain of function of the most abundant smMAF isoform in the liver, MAFG, resulted in preferential repression of lncRNAs in the liver.

### Hepatic MAFG links energy states to glucose metabolism

**Fig. 3:**
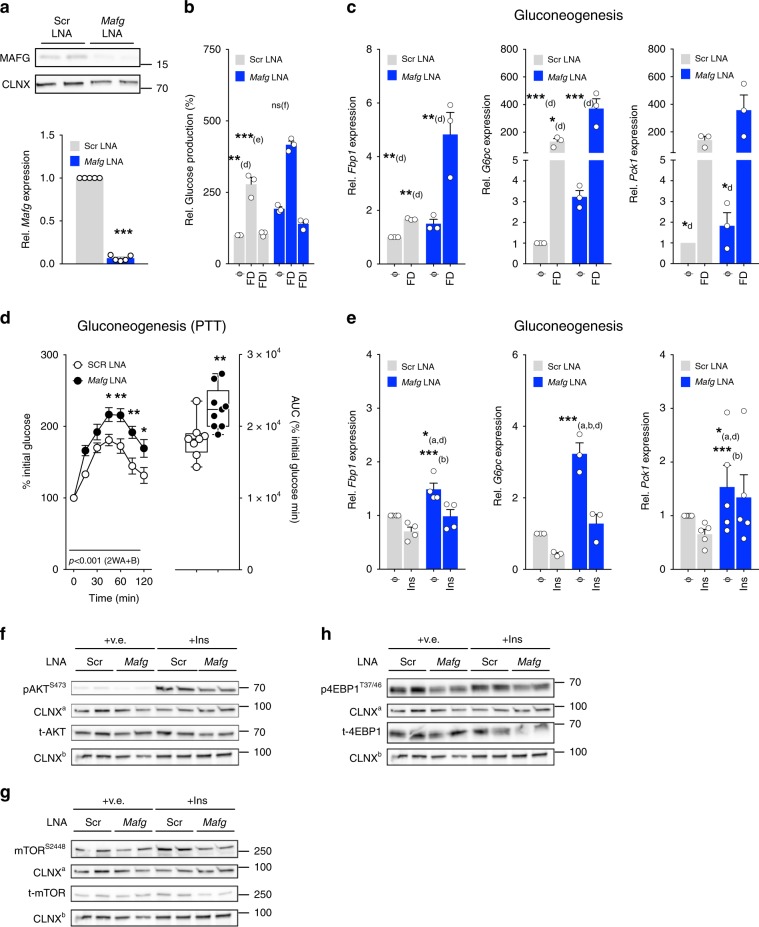
MAFG signaling in hepatocytes represses glucose production and supports mTOR activation. **a** Representative MAFG immunoblot and *Mafg* expression after transfection of primary hepatocytes with 100 nM *Mafg* or Scr LNA. **b** Relative glucose levels in primary hepatocytes after transfection with 100 nM *Mafg* or Scr LNAs and stimulation for 24 h with 10 µm Fsk plus 100 nM Dex (FD) or FD combined with 100 nM insulin (FDI). **c**, **e** Indicated mRNAs after transfection with *Mafg* or Scr LNAs under basal conditions or stimulated with **c** 10 µM Fsk plus 100 nM Dex (FD) or **e** 100 nM insulin. Data represent **a** *n* = 5 or **b**, **c**, **e** *n* = 3–4 independent experiments, each performed in triplicates. **d** Pyruvate tolerance test (PTT) performed in C57BL/6N mice after 6 weeks of HFD feeding with 10 mg kg^−1^ of *Mafg* (*n* = 9) or Scr (*n* = 8) LNA injected 5 days before. **f**–**h** Immunoblots of **f** total and Serine 473 phosphorylated AKT/PKB (pAKT^S473^). **g** Total and Serine 2448 phosphorylated mammalian target of rapamycin (pmTOR^S2448^) and **h** total and Threonine 37/46 phosphorylated eukaryotic translation initiation factor 4E binding protein 1 (p4EB-BP1^37/46^) after transfection of primary hepatocytes with 100 nM *Mafg* or Scr LNAs and stimulated for 10 min with vehicle (*v.e*.) or 100 nM insulin. Separate membranes were loaded with equal amounts of protein lysate for total and phospho-specific immunoblotting, and equal loading was confirmed by calnexin (CLNX) for each membrane. Due to different molecular weights of mTOR, PKB/AKT, and 4E-BP1, these proteins were partly investigated on the same membranes together with the same CLNX antibody. CLNX^a^ and CLNX^b^ bands are therefore identical. Each blot is representative of *n* = 3 immunoblots, each immunoblot performed after transfection of *Mafg* vs. Scr LNA in hepatocyte preparations from *n* = 2 mice. **a**–**c**, **e** Bar graphs represent mean ± s.e.m. with all data points plotted and statistical differences were calculated using **a** paired, two-tailed Student’s *t* test. **b**, **c**, **e** One-way ANOVA and **d** two-way ANOVA with Bonferroni post correction for multiple testing. Superscripts depict group comparisons for post analysis (a–f = comparison vs. column 1–6). **d** Box plot bounds depict upper and lower quartiles with median as the center. Whiskers span all values: *p < 0.05, **p < 0.01, ***p < 0.001. Source data are provided as a Source Data file.

SmMAFs recruit Cap’n’Collar protein like NFE2L1/2 (or NRF1/2) to antioxidant response elements of xenobiotic enzymes^43^ to govern bile acid homeostasis^40^ and hepatic lipid and amino acid metabolism^44^. To determine if MAFG is required to mediate metabolic changes during DIO, we first used an in vitro approach: using LNA transfection, we silenced *Mafg* in primary hepatocytes and observed robust suppression of *Mafg* mRNA and protein (Fig. 3a). To mimic fasting or RF in vitro, we treated cells with Forskolin plus Dexamethasone (FD) or FD combined with insulin (Ins, termed FDI), as reported^45^. At baseline, silencing *Mafg* increased glucose production (Fig. 3b). Treating cells with FD increased glucose production as expected, yet increases in glucose production remained higher when *Mafg* was silenced, suggesting increased gluconeogenesis. Importantly, no in vitro differences in hepatic insulin sensitivity were observed, as insulin treatment reduced glucose production in control (Scr) and *Mafg* LNA- treated cells to similar extents (Fig. 3b). In line with increased glucose production, *Mafg* RNAi increased basal gluconeogenic gene expression as evidenced by elevated fructose-bisphosphatase 1 (Fbp1), glucose-6-phosphatase catalytic subunit (G6pc), and phosphoenolpyruvate carboxykinase 1 (Pck1) expression, and when cells were stimulated with FD, *Fbp1* and *G6pc* remained markedly increased (Fig. 3c). This increased glucogenic tone was confirmed by silencing MAFG using *Mafg* or control Scr LNAs in C57BL/6N mice. MAFG RNAi increased relative glucose production during a PTT, providing in vivo evidence of elevated gluconeogenesis (Fig. 3d). Importantly, LNA-mediated silencing of other smMAFs (*Maff* and *Mafk*) did not increase glucogenic gene expression (Supplementary Fig. 2h–j), emphasizing specific roles for MAFG in controlling hepatic glucose output. In line with unaltered suppression of glucose production (Fig. 3b), insulin-mediated suppression of gluconeogenic enzymes under basal but also FD-stimulated conditions was unchanged after *Mafg* loss, again supporting the notion that *Mafg* loss of function does not affect insulin sensitivity (Fig. 3e). Consequently, when performing Western Blot analysis in insulin-stimulated hepatocytes in the presence or absence of *Mafg*, we observed modest reductions in phosphorylation of serine 473 residue of serine/threonine kinase AKT/PKB (Fig. 3f), yet reduced phosphorylation of the serine 9 of glycogen synthase kinase 3 beta (GSK3B, Supplementary Fig. 2k), which is dispensible for HGP^46^. This suggested that IR-proximal kinases controlling glucose output, but not more distal events, are not affected by *Mafg* loss.

Another important TF pathway that translates information about nutrient states into increases in cell proliferation and protein translation is mammalian target of rapamycin (mTOR)^47^: we found insulin-evoked induction of serine 2448 mTOR phosphorylation (Fig. 3g) and concomitant increases in mTOR Complex 1 (mTORC1) activity, as evidenced by mTORC1-dependent threonine 37/46 phosphorylation of eIF4E-binding protein 1 (p4E-BP1, Fig. 3h)^48^, blunted after *Mafg* silencing, suggesting that MAFG supports mTORC1-dependent processes like ribosome biogenesis and cap-dependent translation^49^ after insulin stimulation. Thus, we showed that increases in MAFG signaling (e.g., in DIO and after RF) preferentially repressed lncRNAs in primary hepatocytes, while LNA-mediated loss of *Mafg* induced lncRNAs, elicited a fasting-like expression profile, and caused specific defects in insulin-dependent activation of signaling pathways linked to protein translation.

### MAFG loss protects from obesity-induced hyperglycemia

**Fig. 4:**
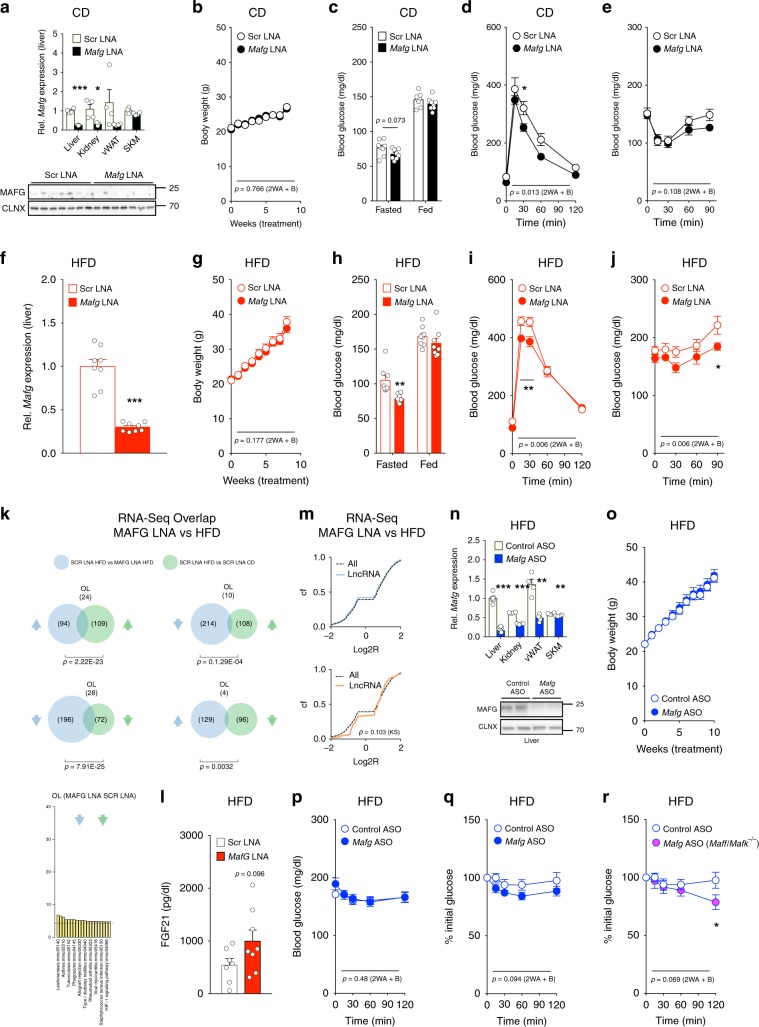
MAFG loss protects from obesity-induced hyperglycemia. **a** qPCR of *Mafg* expression in the liver from CD-fed 21-week-old C57BL/6N mice after 15 weeks of 10 mg kg^−1^ of *Mafg* (*n* = 8) or Scr LNA (*n* = 7) and immunoblot of MAFG and CLNX protein. **b** Body weight, **c** 16-h fasted, and random-fed glucose, **d** GTT, **e** ITT in CD-fed C57BL/6N mice after 6 and 5 weeks of *Mafg* or Scr LNA (*n* = 8). **f** qPCR of *Mafg* expression in the liver from 21-week-old HFD-fed mice after 15 weeks of *Mafg* or Scr LNA (*n* = 8). **g** Body weight, **h** 16-h fasted, and random-fed glucose, **i** GTT, and **j** ITT in HFD-fed C57BL/6N mice after 6 and 5 weeks of *Mafg* or Scr LNA (*n* = 8). **k** DGE overlap and enriched categories in livers from CD- or HFD-fed C57BL/6N mice after 10 weeks of *Mafg* or Scr LNA (*n* = 4). **l** FGF21 levels from 21-week-old HFD-fed C57BL/6N mice after 15 weeks of *Mafg* (*n* = 8) or Scr LNA (*n* = 7). **m** Log2R Cf of mRNA and lncRNA expression changes in 21-week-old HFD-fed mice after 15 weeks of *Mafg* or Scr LNA (*n* = 3). **n** qPCR of *Mafg* expression in liver and indicated tissues after 20 weeks of 5 mg kg^−1^ *Mafg* or Control ASO (*n* = 7 *Mafg* and *n* = 10 Control ASO in liver, *n* = 4 other tissues) and immunoblot of MAFG and CLNX in the liver of HFD-fed C57BL/6N mice after 2 days of *Mafg* or Control ASO. **o** BW in 16-week-old HFD-fed mice after 10 weeks of *Mafg* or Control ASO (*n* = 7 *Mafg* and *n* = 10 Control ASO). Absolute **p** relative **q**, **r** glucose during ITT in HFD-fed **p**, **q** C57BL/6N or **r** *Maff/Mafk*^*−/−*^ (DKO) mice after 11 weeks of *Mafg* or Control ASO (*n* = 6 Control ASO, *n* = 9 *Mafg* ASO, and *n* = 6 *Mafg* ASO in DKO). Bar graphs represent mean ± s.e.m. with all data plotted and statistical differences calculated using **a**, **c**, **f**, **h**, **n** unpaired, two-tailed and **l** unpaired, one-tailed Student’s *t* test. **m** Kolgomorov–Smirnov **b**, **d**, **e**, **g**, **i**, **j**, **p**–**r** two-way ANOVA with Bonferroni post correction. **k** Significance of gene overlaps was calculated by using a hypergeometric distribution: *p < 0.05, **p < 0.01, ***p < 0.001. Source data are provided as a Source Data file.

Having identified MAFG as an important regulator of lncRNA expression, and showing that *Mafg* repressed glucose output in hepatocytes, we next turned to in vivo loss of function. Given the obesity-evoked increase in MAFG signaling, we hypothesized that *Mafg* loss in vivo could be metabolically favorable in DIO and conducted *Mafg* knockdown by performing biweekly injections of *Mafg* or Scr LNAs in CD- or HFD-fed C57BL/6N mice. *Mafg* treatment was well tolerated over the duration of 12 weeks and caused suppression of *Mafg* mRNA and protein expression in lean and obese liver (Fig. 4a, f) and kidney (Supplementary Fig. 3a), but did not affect liver *Maff* or *Mafk* expression in the liver (Supplementary Fig. 3b). When studying the metabolic consequences of *Mafg* silencing, we did not observe alterations in body weight (BW) in CD (Fig. 4b) or HFD-fed (Fig. 4g) mice. *Mafg* knockdown modestly reduced fasting blood glucose (*p* = 0.073), but did not have an effect on blood glucose concentrations in the fed state (Fig. 4c), improved glucose tolerance (Fig. 4d), and trended toward improved insulin sensitivity (Fig. 4e) in CD-fed mice with more pronounced effects in obese animals (Fig. 4h–j). Indirect calorimetric quantification of oxygen consumption, carbon dioxide production, respiratory exchange ratios, energy expenditure, and food intake revealed no differences between groups on either CD or HFD (Supplementary Fig. 3c–g). No histological changes between genotypes were observed concerning fat accumulation in obese livers (Supplementary Fig. 3h). Collectively, these results implicated hepatic alterations in glucose homeostasis in the observed improvement of metabolism in *Mafg* LNA-treated mice. When performing RNA-Seq analysis in CD-fed Scr LNA-treated vs. HFD-fed Scr LNA-treated mice, we observed 218 genes significantly altered. When we compared these gene changes to the 357 genes significantly changed by *Mafg* LNA vs. Scr LNA treatment in HFD-fed mice, we found *n* = 66 genes shared between both groups (Fig. 4k and Supplementary Data 8 and 12). Interestingly, we found that genes were induced by DIO as often reduced by *Mafg* LNA treatment. Particularly the DIO-associated increase in inflammatory gene categories was repressed in *Mafg*-deficient obese livers (Fig. 4k). These transcriptome analyses suggested that *Mafg* inhibition reinstated a healthy, less inflammatory expression profile in livers of obese mice, exemplified by trends toward increased serum fibroblast growth factor 21 (FGF21) levels (Fig. 4l), a hepatokine beneficial in the context of obesity in mice^50^. Furthermore, we observed trends (*p* = 0.103) toward global lncRNA derepression (Fig. 4m) as expected from *Mafg* RNAi in vitro.

To corroborate our findings of improved metabolic control upon *Mafg* silencing during obesity using independent approaches, we repeated our RNAi studies using N-acetyl galactosamine (GalNAc)-conjugated *Mafg* and Control antisense oligonucleotide (ASO) inhibitors. In addition, given the presence of three, presumably functionally redundant^51^, smMAF isoforms in the liver, we silenced *Mafg* in the liver of HFD-fed C57BL/6 or global MafF^−/−^/MafK^−/−^ double-knockout (DKO) mice. After 2 weeks of initial ASO administration, mice were fed HFD for 18 weeks. As for LNA-evoked *Mafg* ablation, *Mafg* ASO sustainably reduced *Mafg* mRNA and protein not only in the liver, but also in the kidney, vWAT, and SKM (Fig. 4n), yet did not affect *Maff* and *Mafk* expression (Supplementary Fig. 3i). Also similar to our previous study by using *Mafg* LNAs, in the absence of overt BW changes (Fig. 4o), *Mafg* loss improved insulin sensitivity (Fig. 4p, q), yet no changes in glycemia, insulin, and triglyceride levels were observed (Supplementary Fig. 3j). Crucially, ablating *Mafg* upon preexisting *Maff*/*Mafk* deficiency resulted in further improvements in insulin sensitivity (Fig. 4r). Thus, we here demonstrate that *Mafg* loss using two independent RNAi approaches improved glucose metabolism and insulin sensitivity, particularly in obese mice.

### LincIRS2 knockdown causes insulin resistance in lean mice

**Fig. 5:**
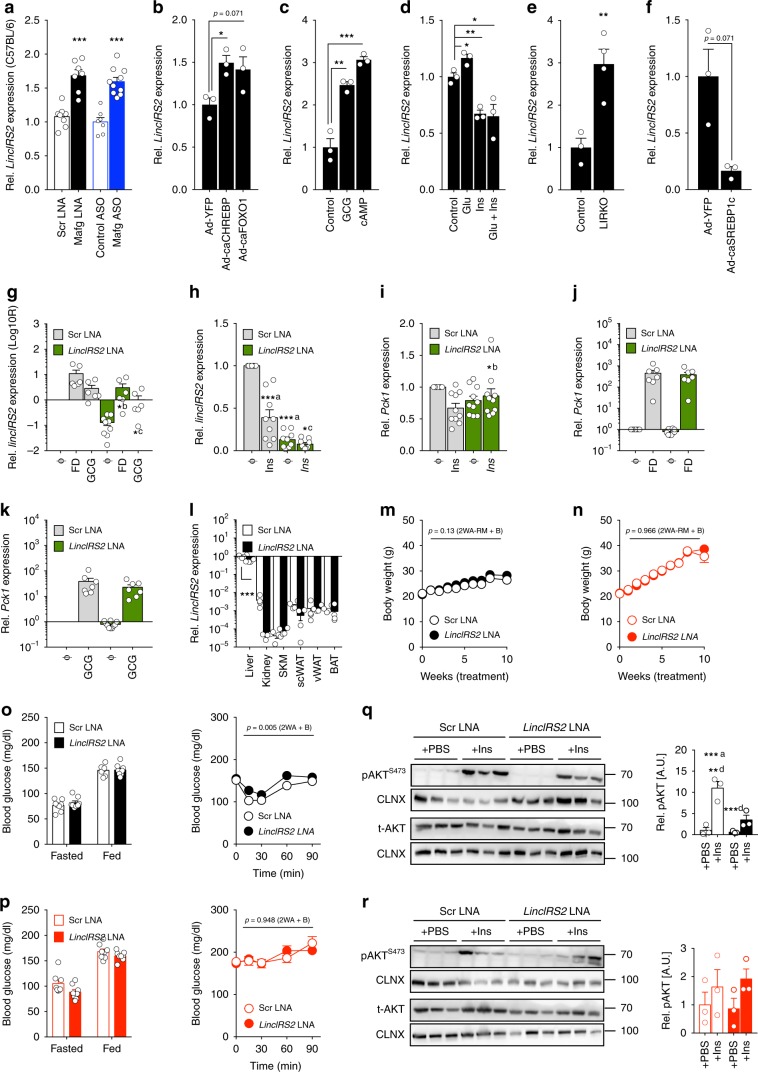
Knockdown of MAFG-dependent LincIRS2 causes mild insulin resistance in lean mice. **a** qPCR of *LincIRS2* expression in the liver from C57BL/6N mice treated with Scr LNA (*n* = 7), *Mafg* LNA (*n* = 8), Control ASO (*n* = 10), or *Mafg* ASO (*n* = 7). **b**–**f** qPCR for *LincIRS2* expression in primary hepatocytes after **b** 24 h of transduction with adenoviruses (Ad)-expressing caChREBP and caFOXO1, **c** 16 h of 200 nM GCG and 100 µM cAMP, **d** 6 h of 27.5 mM glucose, and/or **e** 100 nM insulin in livers from mice with hepatocyte-specific IR knockout vs. Cre-negative Controls^59^ (*n* = 3). **f** Twenty-four hours after transduction of primary hepatocytes with Ad-caSREBP1c. **g** qPCR of *LincIRS2* expression after transfection of primary hepatocytes with 100 nM *LincIRS2* or Scr LNAs and 6 h of stimulation with **g** FD and GCG or **h** insulin. **g**, **h** *n* = 6–10 independent experiments, each in triplicate. **i–k** qPCR of *Pck1* expression after transfection with 100 nM *LincIRS2* or Scr LNAs and 6 h of stimulation with **i** insulin, **j** FD, or **k** GCG. **i**–**k** *n* = 5–10 experiments, each in triplicates. **l** qPCR of *LincIRS2* expression in indicated tissues from 21-week-old CD-fed mice after 15 weeks of 10 mg kg^−1^ *LincIRS2* or Scr LNA (*n* = 4) treatment. **m**, **n** Body weight in **m** CD- and **n** HFD-fed C57BL/6N mice during 15 weeks of *LincIRS2* LNA or Scr LNA (*n* = 8) treatment. **o** Fasted and random-fed GTT and ITT in CD-fed 12- and 11-week-old C57BL/6N mice after 6 and 5 weeks of *LincIRS2* or Scr LNA (n = 8) treatment. **p** Fasted and random-fed glucose and ITT in HFD-fed 12- and 11-week-old C57BL/6N mice after 6 and 5 weeks of *LincIRS2* or Scr LNA (*n* = 8 each) treatment. **q**, **r** Representative immunoblot and densitometry of t-AKT and pAKT^S473^ in **p** CD- or **r** HFD-fed C57BL/6N mice after 15 weeks of *LincIRS2* or Scr LNA treatment injected with 2.5 µl g^−1^ BW NaCl 0.9% or 0.1 U/mouse insulin (*n* = 3). Bar graphs represent mean ± s.e.m. with all data plotted and statistical differences calculated using (**a**–**i**), **l** unpaired, two-tailed Student’s *t* test. **q** One-way ANOVA or **m**–**p** two-way ANOVA with Bonferroni post correction for multiple testing. *p < 0.05, **p < 0.01, ***p < 0.001. Source data are provided as a Source Data file.

Having demonstrated that loss of MAFG not only improved glucose homeostasis and insulin sensitivity but also, concurrently, controlled lncRNA abundance in the liver, we asked if MAFG-repressed lncRNAs are implicated in liver metabolism focused on characterizing MAFG-dependent L-DIO-lncRNA *4833411C07Rik*, which we refer to as “*LincIRS2*” due to its positioning 80 kb 5′ of *Irs2* (Supplementary Fig. 4a) in the following. As expected from our previous *Mafg* overexpression data, *LincIRS2* was induced in livers from *Mafg* LNA and *Mafg* ASO-treated C57BL/6N mice (Fig. 5a). As obesity represents a complex scenario of dysregulated glucose, insulin, lipid, and hormone homeostasis that all affect liver energy metabolism^1^, we investigated which obesity-related nutrients, hormonal factors, and nutrient-sensitive TFs, in addition to MAFG, regulated *LincIRS2*. For this, we expressed constitutively active (ca) versions of metaboregulatory TFs in primary hepatocytes, including glucose-sensing MLX-interacting protein like/carbohydrate response element-binding protein (caMLXIPL/ChREBP^52^), FOXO1 (caFOXO11^53^), or lipogenic caSREBP1C^54^. We found that *LincIRS2* was induced upon catabolic caFOXO1 and caChREBP expression in primary hepatocytes (Fig. 5b) in addition to fasting–mimicking cyclic adenosin-3′,5′-monophosphate (cAMP) and glucagon (GCG) stimulation (Fig. 5c). On the other hand, insulin (with or without glucose co-stimulation) repressed *LincIRS2* (Fig. 5d), whereas IR knockout in liver induced *LincIRS2* (Fig. 5e). Concomitantly, constitutive active versions of (insulin-dependent) SREBP1C (caSREBP1) decreased *LincIRS2* expression (Fig. 5f). Thus, *LincIRS2* inversely correlates with MAFG and insulin signal transduction across several in vitro and in vivo models of altered signaling and nutritional states.

We next asked if, like MAFG, *LincIRS2* is involved in hepatic glucose control. LNA-mediated *LincIRS2* knockdown in primary hepatocytes was highly efficient under basal, FD, GCG, and insulin-stimulated conditions (Fig. 5g, h). Crucially, *LincIRS2*-deficient primary hepatocytes did not exhibit suppression of *Pck1* expression upon insulin stimulation (Fig. 5i). These data suggested in vitro insulin resistance upon *LincIRS2* silencing as, in contrast to *Mafg*, *LincIRS2* RNAi did not increase *Pck1* expression under basal, FD, and GCG stimulation (Fig. 5j, k).

We next investigated the consequences of *LincIRS2* knockdown in vivo by performing biweekly injections of LNAs targeting *LincIRS2*. *LincIRS2* silencing reduced 50% of liver and kidney *LincIRS2* in lean mice (Fig. 5l), and again, we observed no differences in BW between genotypes in CD- or HFD-fed mice (Fig. 5m, n). *LincIRS2* LNA-treated lean mice were normoglycemic (Fig. 5o, *left*), yet mildly insulin resistant (Fig. 5o, *right*), whereas *LincIRS2* LNA-treated obese mice were unaffected, presumably due to preestablished insulin resistance in these animals (Fig. 5p). In line with this, *LincIRS2* RNAi reduced insulin-mediated AKT/PKB phosphorylation of serine 473 in livers from lean (Fig. 5q) but not obese (Fig. 5r) mice in the absence of changes to glucose tolerance (Supplementary Fig. 4b) or hepatic lipid accumulation (Supplementary Fig. 4c). RNA-Seq analysis of *LincIRS2* LNA-treated livers confirmed the deterioration of metabolism at the transcriptome-wide level as DGE in *lincRS2*-deficient lean mice revealed similar gene sets affected as in Scr LNA-treated obese mice (Supplementary Fig. 4d, Supplementary Data 7 and 9).

### Knockout of LincIRS2 causes elevated glucose levels in mice

**Fig. 6:**
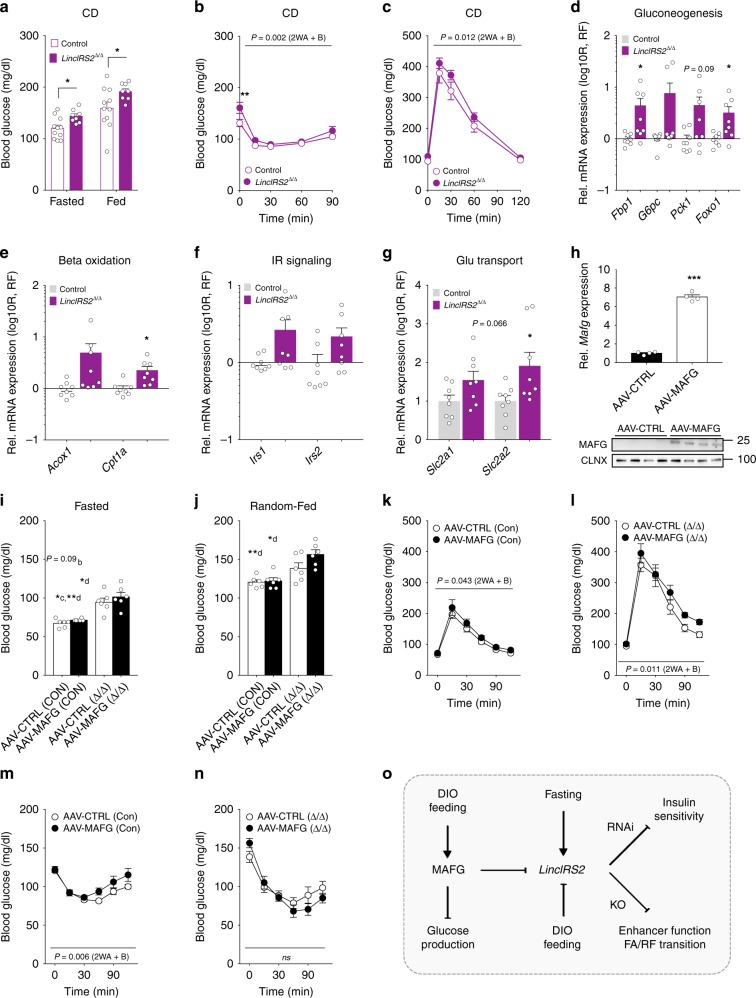
CRISPR-mediated deletion of LincIRS2 impairs glucose metabolism in lean mice. **a** Fasted and random-fed glucose in CD-fed 12-week-old *LincIRS2*^*∆/∆*^ mutant mice (*n* = 8) vs. wild-type littermates (*n* = 11). **b** ITT in 11-week-old and **c** GTT in 12-week-old, CD-fed *LincIRS2*^*∆/∆*^ (*n* = 8) vs. Control mice (*n* = 11). **d**–**g** qPCR of **d** gluconeogenic **e** oxidative **f** IR signaling and **g** glucose transporter expression in 8–15-week-old *LincIRS2*^*∆/∆*^ vs. *LincIRS2*^*∆/wt*^ (*n* = 8) mice. **h** Hepatic qPCR and immunoblot (*n* = 4) from 18- to 20-week-old C57BL/6N 10 weeks after i.v. injection with 1× 10E11 genomic copies/animal encoding AAV8-CTRL or AAV8-MAFG. Fasted **i** and random-fed **j** glucose. **k**, **l** GTT and **m**, **n** ITT in CD-fed 18–20-week-old *LincIRS2*^*∆/∆*^ vs. C57BL/6N Controls (*n* = 12 each) with half of mice in each genotype (*n* = 6) i.v. injected with 1× 10E11 genomic copies/animal AAV8-CTRL or AAV8-MAFG. **o** Illustration of MAFG and *LincIRS2* in regulation of hepatic glucose metabolism. Bar graphs represent mean ± s.e.m. with all data plotted and statistical differences were calculated using **a**, **d**–**h** unpaired, two-tailed Student’s *t* test. **i**, **j** One-way ANOVA with Bonferroni post correction for multiple testing. **b**, **c**, **k**–**n** Two-way ANOVA with Bonferroni post correction for multiple testing. Superscripts depict group comparisons for post analysis (a–f = comparison vs. columns 1–6) *p < 0.05, **p < 0.01, ***p < 0.001. Source data are provided as a Source Data file.

Given the mild deterioration of metabolism upon transient *LincIRS2* knockdown in LNA-treated mice, we wanted to address the role of *LincIRS2* in glucose metabolism using a genetic model. For this, we employed CRISPR/Cas9 genome editing in order to target a 418-bp region encompassing exon 1 and the putative promoter region (marked by H3K4me3 and H3K9Ac, Supplementary Fig. 4f–i) of *LincIRS2*. Sanger sequencing of the *LincIRS2* allele confirmed a 426-bp deletion, together with a 5-bp insertion of the desired genomic *LincIRS2* locus (Supplementary Fig. 4j). Homozygous C57BL/6N-*LincIRS2*^*em/Cecad*^ knockout mice (termed *LincIRS2*^*∆/∆*^) exhibited complete absence of *LincIRS2* expression (Supplementary Fig. 4k), were born at Mendelian frequencies, and exhibited no developmental or behavioral abnormalities, changes in BW, energy expenditure, or substrate mobilization (Supplementary Fig. 4l–n). Lean *LincIRS2*^*∆/∆*^ mutants exhibited elevated blood glucose levels under fasted and fed conditions (Fig. 6a) and glucose levels remained high after insulin (Fig. 6b) or glucose challenge (Fig. 6c). Interestingly, *LincIRS2*^*∆/∆*^ mice showed elevated gluconeogenic *G6pc*, *Pck1*, and *Foxo1* (Fig. 6d), oxidative *Acyl-CoA Oxidase 1* (*Acox1*) and *Carnitine Palmitoyltransferase 1A* (*Cpt1a*, Fig. 6e), *insulin receptor substrate* (*Irs*) 1 and 2 (Fig. 6f), and *Slc2a1-2* glucose transporter expression (Fig. 6g), suggesting altered transcriptional regulation of these enzymes underlying the elevated glycemia in these mice.

Finally, and to test if *LincIRS2* synergized with MAFG function, we overexpressed *Mafg* using adeno-associated viruses in C57BL/6 and *LincIRS2∆/∆* mice. Adeno-associated virus serotype 8 (AAV8)-mediated overexpression (AAV8-MAFG) increased *Mafg* mRNA and protein (Fig. 6h), but did not alter fasted or random-fed glucose levels (Fig. 6i, j). Intriguingly though, driving *Mafg*, alone or coupled to *LincIRS2 loss*, again caused glucose intolerance to similar extents, arguing for synergistic roles of MAFG overexpression and *LincIRS2* deletion (Fig. 6k, l) in glucose metabolism. Conversely, delivery of *Mafg* alone caused insulin resistance (Fig. 6m), but not when coupled to *LincIRS2* loss (Fig. 6n), suggesting independent roles in regulating insulin sensitivity. Taken together, our data identified a transcriptional pathway where MAFG acts as a repressor of metabolically regulated hepatic lncRNAs. We identified a specific MAFG-repressed lncRNA, *LincIRS2*, that contributed to glucose homeostasis and insulin-mediated suppression of hepatic glucose production in mice. Either genetic *LincIRS2* deletion or *LincIRS2* RNAi in vivo impaired glucose metabolism, albeit to different degrees, and presumably, via different molecular mechanisms.

Collectively, we demonstrated a hitherto unknown role for MAFG in serving as intracellular sensor of systemic nutrient states, where changes in MAFG signal transduction are coupled to alteration of hepatic glucose homeostasis. Further, increased MAFG signaling partly explained the initial observation of opposite regulation of lncRNAs vs. mRNAs during DIO and T2D. One molecular effector mechanism of how excessive MAFG activity during DIO translated into glucose alterations is repression of liver-selective *LincIRS2*, which itself integrated anabolic and catabolic metabolites and TF responses (illustrated in Fig. 6o). In vivo genome editing and RNAi against *LincIRS2* revealed roles for this MAFG-lncRNA axis in ensuring hepatic glucose handling and maintenance of insulin sensitivity.

## Discussion

Our results establish the TF MAFG as a regulator of energy-rich nutrient states in the liver, both in postprandial responses and during metabolic diseases like obesity and type 2 diabetes. Mechanistically, we demonstrated that MAFG controls a specific set of obesity-associated hepatic lncRNAs that are repressed in obese livers, in addition to the known function of MAFG in bile acid regulation^40^.

Our results show little evidence for co-regulation with only four disease-associated syntenically conserved lncRNAs in human and mouse livers (Supplementary Data 13) when performing comparative lncRNA analyses using PLAR^55^. This may be due to (1) misannotation of gene biotypes, as not every lncRNA transcript truly represents a noncoding transcript, evidenced, e.g., by the high protein-coding potential of lncRNA *Gm10319*, (2) due to the fact that lncRNA conservation at the structural and function level is hard to address computationally^56^, or (3) conservation of disease-associated lncRNAs simply is low as shown in other cell types^57^. Another caveat lies in the low number of obesity-associated lncRNAs we identified here. This limits the statistical power of TF motif enrichment algorithms. However, other in silico studies came to similar conclusions in terms of smMAF TFBS enrichment in long noncoding RNA promoters^31,32,35^.

In our study, we found that MAFG signaling activity was reduced during fasting, which has not been previously reported. We then analyzed both MAFG and its known interaction partner NRF1/NFE2L1 during nutrient deprivation and found that, while both *Nrf1/Nfe2l1* and *Mafg* mRNA were mildly induced, NRF1/NFE2L1, but not MAFG protein, was profoundly upregulated during fasting (Supplementary Fig. 5a, b). By reanalyzing publicly available hepatic RNA-Seq data performed in mice with hepatocyte-specific deletion of NRF1/NFE2L1^58^ (GEO ID: GSE103949), we found that NRF1/NFE2L1 ablation resulted in strikingly similar expression profiles compared with MAFG overexpression (Supplementary Fig. 5c). These data suggested that the interaction of MAFG with NRF1/NFE2L1 constitutes an important regulatory step during fasting in the liver: recruitment of MAFG into NRF1/NFE2L1:MAFG heterodimer complexes, for instance after fasting-induced increases in NRF1/NFE2L1 protein would, in turn, reduce MAFG target gene expression (as during fasting), consistent with a notion where NRF1/NFE2L1 deficiency and MAFG overexpression increase pools of “free” MAFG homodimers with known gene-repressive functions^36,37^. Although we have not directly addressed the binding partners of MAFG under different nutrient stress conditions, our data suggest that competition between NRF1/NFE2L1 and MAFG is important for liver physiology: as found for MAFG gain of function^40^, NRF1/NFE2L1 inactivation in the liver downregulated genes involved in cholesterol to bile acid conversion, e.g., *Cyp7b1*^58^. This suggests that, whereas NRF1/NFE2L1 suppresses inflammation and promotes bile acid synthesis^40^, MAFG overexpression induces hepatic inflammation (Fig. 5k) and suppresses bile acid conversion^40^, in addition to suppressing key regulatory lncRNAs such as *LincIRS2* (Supplementary Fig. 5d, e and Supplementary Data 11). Whether the MAFG-dependent regulation of lncRNAs (e.g., *LincIRS2*), or the MAFG-dependent regulation of coding mRNAs are key in mediating the MAFG-dependent changes in energy-rich nutrient states remains to be determined. However, our data suggest that the MAFG-dependent regulation of coding and noncoding mRNAs is complimentary, suggesting that both are required to elicit the full response.

Ultimately, our findings could also indicate that MAFG pathway inhibition, or NRF1/NFE2L1 activation, could generally favor energy-preserving processes where transcription of long noncoding RNAs does not require subsequent mRNA translation. Conversely, postprandial or obesity-associated activation of MAFG would trigger energy-demanding, anaplerotic processes like an increase in cellular proliferation and protein synthesis as a consequence of mTOR-4E-BP1 pathway activation.

## Methods

### Animal care and research diets

All animals were maintained on a C57BL/6N background, housed in groups of 3–5 animals per cage at 22–24 °C on a constant 12-h light/dark cycle in a SPF-controlled facility with regular testing for pathogens. Care of animals and all mouse research was approved by and we adhered to institutional and animal-care committee ethical guidelines from local (Bezirksregierung Köln) or regional (Tierschutzkommission accession no. §15 TSchG of the Landesamt for Natur, Umwelt und Verbraucherschutz (LANUV) North-Rhine Westphalia, Germany, internal reference no. Az-84-02.04-2016.A460) authorities. Upon weaning, mice were fed standard rodent chow (Teklad Global Rodent T.2018.R12; Harlan). For experiments involving controlled feeding paradigms, animals were allowed AL access to CD (D12450B* mod LS; Sniff) containing 62 kJ% carbohydrates, 27 kJ% protein, and 11 kJ% fat and drinking water. DIO was achieved by feeding a HFD (D12492 (I) mod; Sniff) containing 22 kJ% carbohydrates, 24k J% protein, and 54 kJ% fat from starting at 6–7 weeks of age. Experimental mice were exposed to specific diets for 10–12 weeks and 17–18 weeks old at sacrifice unless described otherwise. Six-month-old LIRKO^59^ (Albumin-*Cre*^+/−^, *IR*^*flox/flox*^) and littermate control (Albumin-*Cre*^−/−^, *IR*^*flox/flox*^) animals were used.

### RNA isolation and total RNA-Sequencing

Liver samples used for total RNA-Sequencing (RNA-Seq) were from male, 36-week-old C57BL/6N mice exposed to CD (*n* = 3) or HFD (*n* = 3) feeding for 30 weeks, starting at 6 weeks of age. RNA-Seq of C57BL/6N liver mice exposed to different nutrient states was performed in liver samples from male, 17-week-old, lean AL (*n* = 4), AL followed by 16 h of fasting (FA, *n* = 4), and FA followed by 6 h of RF (*n* = 4) mice. For RNA-Seq from a genetic model of obesity and T2D, male *BKS.Cg-Dock*^*7m+/+*^*; LepR*^*db*^*/J* (*db/db*) mice and *BKS.Cg-Dock*^*7m+/+*^ *(misty/misty)* control mice were purchased from Jackson Laboratory or Janvier Labs. We used *misty* mice as control animals, as they are the littermates of *db/db* mice generated on the BKS.Cg-Dock^7m+/+^ *LepR*^*db*^/J background (*n* = 4 mice per genotype). Tissues were collected at 10 weeks of age. RNA-Seq was also performed in lthe iver from mice treated with adenoviruses expressing *Mafg* cDNA (Ad-MAFG, *n* = 3) or empty vector (Ad-CMV, *n* = 3) under control of mouse cytomegalovirus (CMV) promoter^40^. Briefly, cDNAs for mouse MAFG, were cloned from whole-liver cDNA into pAdTrack CMV plasmids. For animal experiments, 1× 10E9 plaque-forming units were used, and tissues were collected after 5–7 days of viral treatment. RNA-Seq was also performed in the liver from 21-week-old male mice exposed to CD or HFD and injected 15 weeks with control (Scr), *Mafg*, or *LincIRS2* LNA (*n* = 4 per genotype and diet). Human liver samples used for RNA-Seq were obtained from male individuals that were with lean and nondiabetic (*n* = 4; age 60.75 ± 9, body mass index (BMI) 23.52 ± 1.36, Patient IDs: 11_ 0381WSC, 13_1651HRU, 135_1311WER, and 155_3141AZE), obese and nondiabetic (*n* = 4; age 38.5 ± 13.42, BMI 51 ± 2.98, Patient IDs: 31_0311RAN, 32_0861NSC, 37_1941JRO, and 98_0121STR), overweight, diabetic (*n* = 2; age 75.5 ± 0.7, BMI 26 ± 1.41, Patient IDs: 54_0951LZU, 56_1781DGR, 95_1701GBO, and 152_3011FHE), and obese and diabetic (*n* = 2; age 44 ± 2.8, BMI 48.25 ± 20.85). All patients gave written informed consent, and approval of the ethics committee of the University of Ulm was obtained^29^. Library preparation and sequencing was performed at (1) Max-Planck Genome-Centre (MP-GC, HFD vs. CD cohort) or (2) the Cologne Center for Genomics (CCG), Germany (others). Following quality checks, 1 µg of total liver RNA of each sample was (1) depleted for rRNA using NEBNext® rRNA depletion Kit (human/mouse/rat). Library preparation was performed with NEBNext Ultra^™^ Directional RNA Library Prep Kit for Illumina (New England Biolabs) or (2) depleted for cytoplasmic and mitochondrial rRNA with Ribo-Zero Gold (LNA Cohort) or Ribo-Zero and strand-specific library preparation performed using TruSeq RNA Gold Kit from Illumina. All libraries were sequenced on (1) HiSeq2500 in 2 × 100-bp PE or (2) HiSeq 4000 instruments in 2 × 75 PE sequencing mode.

### RNA-Sequencing data processing

1. Mouse RNA-Seq was processed utilizing the GRCm38 assembly of the mouse genome as gene sets from Ensembl release 90^60^. Biotype and gene features were added manually using Ensembl Biomart. The pipeline consists of six steps: (i) barcode and adapter removal using flexbar 3.4.0^61^, (ii) computational rRNA depletion by filtering reads that map to known rRNAs in mice using Bowtie2 2.2.9^62^, (iii) alignment of non-rRNA reads to the mm10 reference genome using STAR 2.6.0c^63^, (iv) transcript assembly using cufflinks followed by (v) cuffmerge, and (vi) cuffdiff performs DGE analysis between experimental conditions via Cufflinks suite 2.2.1^64^.
2. Human RNA-Seq data were analyzed using QuickNGS^65^, version 1.2.2, based on Ensembl release 82. In brief, reads were mapped to GRCm38 assembly of the human genome using Tophat2^66^, version 2.0.10, and reassembled with Cufflinks, version 2.1.1. DGE was analyzed using the DESeq2^67^, version 1.10.1. The results were uploaded to the QuickNGS database and combined with multiple annotations using the biomaRt package version 2.16.0.

### Gene overlap analysis

Overlap of regulated gene sets were performed with “*venneuler*” package for R using genes defined under (1) in the paragraph above. The *p* value of the overlap was calculated using hypergeometric distribution

$$p = \mathop {\sum }\limits_{i = k}^{\min \left( {m,n} \right)} \frac{{\left( {\begin{array}{*{20}{c}} m \\ i \end{array}} \right)\left( {\begin{array}{*{20}{c}} {N - m} \\ {n - i} \end{array}} \right)}}{{\left( {\begin{array}{*{20}{c}} N \\ n \end{array}} \right)}}$$

where *N* is the total number of testable genes, *m* is regulated genes in study A, *n* is regulated genes in study B, and *k* is genes overlapping studies A and B.

### Ingenuity pathway analysis

IR, FOXO1, and SREBP1C/SREBF1 were loaded as seed gene nodes into IPA and expanded into TF-dependent networks by using the “grow” option with molecular interactions limited to “direct” and “downstream” effects. These manually curated pathways were overlaid with DGE information from RNA-Seq in HFD- vs. CD-fed C57BL/6N mice.

### Promoter analyses and motif enrichment analysis

1. For enrichment analyses of known TFBS using AME^33^ functionality of MEME^34^, RNA-Seq data of livers from mice on CD or HFD feeding were aligned to mm10 using STAR^63^, and transcriptome assembly and DGE analyses performed using CuffLinks/CuffDiff^68^, respectively. Significantly altered mRNAs and lncRNAs were defined by *p* value ≤ 0.1 and L2R > 0 or <0. lncRNAs and mRNAs were defined by filtering for biotypes “*bidirectional_promoter_lncRNA*”, “*lincRNA*”, “*sense_intronic*”, “*sense_overlapping*”, “*antisense*”, and “*protein-coding*” using annotations from Ensembl Biomart Version 90. Promoter sequences (defined as −800/+100 bp from TSS) of each biotype class were extracted and enriched TFBS motifs identified using commands “*ame–verbose 1–oc–bgformat 1–scoring avg–method ranksum–pvalue-report-threshold 0.05 db/JASPAR/JASPAR2018_CORE_vertebrates_non-redundant.meme db/MOUSE/uniprobe_mouse.meme*”.
2. For de novo enrichment analyses of TFBS motifs using HOMER, mRNA, and lncRNA promoter sequences from (1) were used as input for HOMER de novo motif analyses (findMotifsGenome.pl) using indicated promoter lists as background. De novo motif analyses were collapsed to motifs known to bind TFs found expressed in at least one condition in the RNA-Seq data (CD/HFD), using the curated motif list from the IMAGE pipeline^69^.

### Chromatin-immunoprecipitation (ChIP) sequencing analysis

Previously published public BLRP ChIP-seq data from livers of mice transduced with adenoviruses overexpressing BLRP-MAFG fusion protein (GEO ID: GSE77559) were used as proxy for MAFG cistromes in the liver. The ChIP-seq data were aligned to mm10 genome using STAR^63^, and BLRP-MAFG peaks were called in each replicate (*n* = 2) using findPeaks function in HOMER^38^. The peak lists were merged using mergePeaks function in HOMER and only peaks called in both replicates were retained for downstream analyses.

### In vivo RNAi using LNAs

RNA-interference (RNAi)-mediated silencing of *Mafg* and *LincIRS2* was performed using custom-made LNA GapmeRs that were designed and synthesized by Exiqon, Denmark to target murine *Mafg* and *LincIRS2* (*Mafg*, *LincIRS2* LNA). A negative control with no homology to known mouse transcripts was used as control LNA. Four-week-old C57BL/6N male mice were obtained from Charles River and acclimated for 2 weeks, followed by intravenous (i.v.) injection of 10 mg kg^−1^ BW LNA dissolved in 0.9% NaCl once every other week for 15 weeks starting from 6 weeks of age. One week after the start of injection series, mice were placed on CD or HFD for 14 weeks. Insulin and glucose tolerance test (ITTs and GTTs) were performed at 6 and 7 weeks of diet, respectively.

### In vivo RNAi using antisense oligonucleotides (ASO)

Six- to seven-week-old male C57BL/6N mice or *Maff/Mafk*-null (double knockout, DKO) mice were injected weekly with 5 mg kg^−1^ Gal-Nac-conjugated *Mafg* or Control ASO at a concentration of 1 mg/mL dissolved in 1× phosphate-buffered saline (PBS), for 20 weeks. Two weeks after the first injection, mice were placed on HFD for 18 weeks. ITT was performed at 9 weeks on diet, and GTT was performed at 10 and 16 weeks on diet.

### Generation of LincIRS2^∆/∆^ knockout mice using CRISPR/Cas9

Generation of a mouse model for genetic *LincIRS2* deficiency (*LincIRS2*^*∆/∆*^) was performed using CRISPR/Cas9 genome editing where a 426-bp deletion (chr8: 10,899,861–10,900,279) and a 5-bp insertion, encompassing the presumed promoter region (defined by occurrence of histone 3 lysine 4 trimethylation (H3K4me3) near the *LincIRS2* TSS in murine liver, based on NIH Epigenomics Roadmap information^70^) and exon 1 of *LincIRS2* (GRCm38/mm10) were excised. To this end, guide RNAs (gRNAs, sequences provided in Supplementary Data 10) were designed using CRISPOR^71^ and gRNAs with highest specificity scores (≥50) chosen, yet gRNAs with predicted off-target effects for 1 or 2 occurring mismatches excluded. After gRNA identification, target-specific CRISPR RNAs (crRNAs) were incubated with generic transactivating crRNAs (tracrRNAs, Integrated DNA Technologies) to form the active gRNA. Next, annealed gRNAs were incubated with Cas9 proteins to obtain functional ribonucleoprotein complexes. Pronuclear injections of CRISPR/Cas9 complexes into fertilized oocytes of C57BL/6N females were performed at the CECAD in vivo Research Facility^72^. Briefly, we employed pronuclear injection of CRISPR/Cas9 components into C57BL/6NRj zygotes, which were then implanted into pseudopregnant RjHan:NMRI females. Synthetic target-specific crRNA and sequence-independent tracrRNA molecules were purchased from commercial distributors. Shortly before injection, crRNAs and tracrRNAs were co-incubated with recombinant Cas9 proteins, and to enhance the efficacy of CRISPR/Cas9 genome editing, we added Cas9-encoding mRNA to the injection mix. Healthy, CRISPR/Cas9-injected oocytes were subsequently implanted into oviducts of pseudopregnant 0.5 postcoital foster females. Finally, offspring born from implanted oocytes was analyzed via DNA sequencing, and in the case of successful CRISPR/Cas9-mediated *LincIRS2* knockout, bred to C57BL/6N mice to create C57BL/6-*LincIRS2*^*em1Cecad*^ transgenic mouse lines. Genotyping primers are available in Supplementary Data 10. For each of the two gRNAs, the top five sites of predicted off-target Cas9 activity were investigated using Sanger Sequencing, and no changes to the GRCm38/mm10 genomic sequence were detected (Supplementary Data 14).

### Adeno-associated virus (AAV)-mediated MAFG overexpression

PscAAV8-MAFG-HA encoding plasmids were produced by replacing luciferase (Luc) coding sequence in pscTTR-luc by the respective sequences. AAV8 was produced in HEK293 cells using the triple-transfection method. Specifically, the AAV helper plasmid pXR8, the adenoviral helper plasmid pXX6, and the vector plasmid (pscTTR-luc, pscAAV-MAFG-HA) were transfected in 1:1:1 molar ratio using CaP method. Forty-eight hours post transfection, cells were harvested and lysed. Following benzonase treatment and a low-speed centrifugation preclearing step, preparation was purified by iodixanol step gradient purification. Genomic titer was determined by qPCR using ITR-specific primers. C57BL/6N and *LincIRS2∆/∆* male mice were 8–10 weeks of age and *i.v*. injected with 10E^11^ vector particles/animal of genomic copies for AAV control (encoding Luc) or AAV-MAFG. Glucose and insulin tolerance test were performed at 8 and 9 weeks after injection.

### Isolation of primary hepatocytes and liver fractionation

Mouse primary hepatocytes were isolated from C57BL/6N mice livers by a classical two-step collagenase perfusion method^73^ with minor modifications as follows: mice were subjected to experiments around 8–12 weeks of age. Under anesthesia, mice were perfused via the portal vein with 50 ml of perfusion medium, followed by digestion with 50 ml of collagenase medium. After liver perfusion, dissociated cells from the liver were filtered through a 70-µm cell filter (BD Falcon) into DMEM, low glucose (Gibco) supplemented with 10% FBS and 1% penicillin–streptomycin (P/S), and centrifuged twice at 50*g* for 3 min to recover the pellet and the supernatant. Hepatocytes were obtained by resuspending the pellet in 30% Percoll and centrifuging at 150*g* for 7 min. For obtaining non-hepatocyte (non-parenchymal) cell fractions, the supernatant was centrifuged at 350*g*, and the resulting cell pellets centrifuged on a 20% (w/vol) Histodenz (Sigma) gradient. For fractionation analyses, both hepatocytes and non-parenchymal fractions were frozen immediately after isolation. For experiments in which hepatocytes were transfected and/or stimulated, cells were attached to six-well collagen I-coated plates (Costar) for 2 h and were grown in P/S-supplemented DMEM, low glucose (Gibco) without FBS overnight; they were then subjected to experimental procedures 24 h after isolation. The media were as follows: Perfusion medium, HBSS (Gibco) without magnesium or calcium and supplemented with 0.5 mM EGTA; collagenase medium, DMEM, low glucose (Gibco) supplemented with 15 mM HEPES and 100 collagen digestion units (CDU) ml^−1^ of collagenase, type IV (Worthington); 90% concentrated Percoll, 100% Percoll (Amersham) diluted with 10× HBSS (Gibco).

### Subcellular fractionation of primary hepatocytes

Subcellular fractions were obtained using freshly isolated primary hepatocytes using Nuclei Isolation Kits: Nuclei EZ Prep according to the manufacturer’s (Sigma Aldrich) protocol. Briefly, 5 × 10^6^ cells were centrifuged (5 min, 300*g*, 4 °C) and supernatants were discarded, whereas cell pellets were resuspended in 10 ml of PBS and centrifuged again (5 min, 300*g*, 4 °C). After removal of supernatants, 4 ml of ice-cold Nuclei EZ Lysis Buffer was added to cell pellets and the reaction tube vortexed briefly and kept on ice for 5 min. Following centrifugation (5 min, 300*g*, 4 °C), supernatants were kept for later analysis (cytoplasmic fraction), while pellets were resuspended in 4 ml of ice-cold Nuclei EZ Lysis Buffer, vortexed briefly, and kept on ice for 5 min. After a final centrifugation (5 min, 300*g*, 4 °C) supernatants were discarded and nuclei pellets resuspended in 200 µl of ice-cold Nuclei EZ Storage Buffer (nuclear fraction). If not directly processed for RNA isolation, cytoplasmic and nuclear fractions were stored at −80 °C.

### LNA-mediated gene knockdown of primary hepatocytes

Primary hepatocytes from C57BL/6N mice were cultured for 24 h at 37 °C and 5% CO_2_ before transfection of control (Scr), *Mafg*, and *LincIRS2 (4833411C07Rik)* LNAs (sequences provided in Supplementary Data 10). Lipofectamine RNAiMax was diluted 1:16 in DMEM. For a final concentration of 100 nM, the respective LNAs (stock 10 µM) were diluted 1:100 in DMEM. Both solutions were incubated for 5 min at room temperature. LNA and Lipofectamine solutions were mixed at equal volumes and incubated for 5 min. Cells were washed with prewarmed 1× PBS and 1.6 ml of DMEM without FBS added to each well. Four-hundred microliters of LNA/Lipofectamine mix was added and cells incubated for 24 h at 37 °C and 5% CO_2_ before changing the medium. For in vitro stimulation experiments, primary hepatocytes were washed with prewarmed 1× PBS and fresh medium added. Cells were stimulated with 100 nM Insulin (Sigma), Glucagon (0.1 mg ml^−1^, Sigma), 10 µM Forskolin plus 100 nM Dexamethasone (FD), or FD plus 100 nM Insulin (FDI), and incubated for 10 min, 6 h, or 24 h at 37 °C and 5% CO_2_, before harvesting cells for further experimental analysis.

### Quantification of glucose production

After 24 h of transfection, primary hepatocytes were washed with warm 1× PBS, and glucose-free DMEM (Gibco) supplemented with 100 mM sodium pyruvate was added in order to study cell-intrinsical glucose production. Stimulation with Fsk/Dex (10 µM/100 nM) with/without 100 nM insulin was performed for 24 h at 37 °C and 5% CO_2_. Supernatant medium of the cells was collected to measure endogenous glucose production with Glucose-Glo Assay Kits (Promega) following the manufacturer’s instructions.

### Adenoviral overexpression of metabolic TF in hepatocytes

Adenoviruses encoding YFP (Addgene plasmid #15302), constitutively active mouse FOXO1 (caFOXO1, Addgene plasmid #17547), and constitutively active mouse SREBP1c (caSREBP1C, Addgene plasmid #8883) were subcloned from plasmids (Addgene) into the adenoviral vector system pAd/CMV/V5-DEST^™^ (Invitrogen). A constitutively active mouse ChREBP lacking the N-terminal low-glucose inhibitory domain was PCR amplified from mouse liver cDNA^74^ and cloned into pAd/CMV/V5-DEST. Adenoviruses were amplified in HEK293A cells and purified by CsCl gradient centrifugation. Purified viruses were desalted with PD10 columns (GE Healthcare Life Sciences) and were titered with an Adeno-X Rapid Titer Kit (Clontech). Primary hepatocytes were treated with adenoviruses encoding aforementioned TF for indicated times. For in vitro stimulation experiments, cells were exposed to 100 µM cAMP or 200 nM GCG and incubated for 6 h or stimulated with 100 nM Insulin (Sigma) for 16 h, at 37 °C and 5% CO_2_, before harvesting cells for experimental analysis.

### Immunoblot analysis

For protein isolation, primary hepatocytes grown on six-well plates were washed gently one time with ice-cold 1× PBS and excess 1× PBS aspirated. Five-hundred microliters of RIPA lysis buffer (50 mM Tris-HCL, pH 7.5, 150 mM NaCl, 1 mM EDTA, 0.1% sodium deoxycholate, 1% NP-40, 1× protease inhibitor (Sigma), and 1× phosphatase inhibitor (Roche)) was added to each well of the six-well plate and cells were scraped. Protein lysates were transferred to 1.5-ml Eppendorf tubes, snap-frozen in liquid nitrogen, and thawed on ice for three repeated cycles. Tissues were homogenized in 1 ml of RIPA Buffer using FastPrep 24G (MPBio). After 10 min of centrifugation at 12,000*g* and 4 °C, the supernatant was collected into fresh tubes, and protein concentration was determined using the Bradford colorimetric protein method (Thermo Fisher Scientific). Protein samples were stored at −80 °C. Samples for immunoblotting were separated by sodium dodecyl sulfate polyacrylamide gel electrophoresis after being prepared with 4× Laemmli Sample Buffer containing 10% β-mercaptoethanol and heated to 95 °C for 5 min. Subsequently, proteins were transferred to nitrocellulose membrane and blocked with Western Blocking Reagent (Roche). Membranes were incubated with primary antibodies at 4 °C overnight. After incubation with secondary antibodies, blots were developed with Pierce ECL Western Blotting substrate (Thermo Fisher Scientific). All densiometric measurements were performed with ImageJ. Band intensity was normalized to calnexin (CLNX), which served as internal loading control, blotted on the same gel and membrane. Antibodies were purchased from Cell Signaling Technology and raised against pAKT^S473^ (Catalog No. 9271), t-AKT (Catalog No. 4685), pTOR^S2448^ (Catalog No. 2971), t-mTOR (Catalog No. 2972), p4E-BP1^T37/46^ (Catalog No. 2855), t-4E-BP1 (Catalog No. 9452), pGSK3B^S9^ (Catalog No. 9323), t-GSK3B (Catalog No. 9315), and NRF1/NFE2L1 (Catalog No. 8052). MAFG antibody was purchased from GeneTex (GTX 114541). Primary antibodies were used at a 1:1000 dilution ratio in Tween TBS. CLNX antibody was used at a 1:5,000 dilution and purchased from Calbiochem (Catalog No. 208-880).

### Glucose, insulin, and PTT

At the time of performing insulin tolerance tests (ITT), mice were 11 weeks of age and either 4 weeks exposed to CD or HFD feeding. ITT was carried out in random-fed mice in the morning. After determining basal blood glucose levels, each animal received 0.75 IU per kg BW of insulin (Insuman Rapid; Sanofi-Aventis). For glucose tolerance tests (GTT), mice were 12 weeks of age and 5 weeks exposed to CD or HFD feeding. After 16 h of fasting and measurement of basal blood glucose levels, animals were i.p. injected with 2 g per kg BW of glucose (20% glucose, Delta select). Blood glucose levels were recorded using an automatic glucose monitor (Contour, Bayer Diabetes Care) at the indicated time points in (1) male Scr vs. *Mafg* and *LincIRS2* LNA-treated mice, (2) Control ASO vs. *Mafg* ASO, and (3) *LincIRS2*^wt^/^wt^ (Control for *LincIRS2*^∆/∆^) and *LincIRS2*^∆/∆^ mice. In the ASO cohort, animals that showed no decrease of blood glucose after insulin gavage throughout all measurements were excluded from ITT analysis, assuming injection of insulin outside of the peritoneal cavity as required for the assay. The pyruvate tolerance test (PTT) was performed in HFD-fed animals after 6 weeks of HFD feeding with Scr and *Mafg* LNA injection 5 days before the experiment. For the PTT itself, 4 g of pyruvate was dissolved in 20 ml of 0.9% NaCl and injected 10 µl g^−1^ BW after 16 h of fasting (final dose 2 g pyruvate kg^−1^).

### Indirect calorimetry (PhenoMaster)

Upon indirect calorimetry measurements, mice of indicated genotypes and diets were 16 weeks of age and 10 weeks exposed to CD or HFD. All measurements were performed using a PhenoMaster System (TSE Systems), which allowed in-cage metabolic and activity monitoring. Three days before analysis, mice were placed alone in training cages, identical to the 7.1-l chambers of the PhenoMaster open-circuit calorimetry system and continued to receive the respective diets (CD and HFD) throughout training and data acquisition. Diets and water were provided AL in the appropriate devices, and food intake measured by the built-in automated instruments. Parameters of indirect calorimetry were measured initially for 96 h at 22 °C and mean values calculated for each time of day.

### Tissue collection

At the end of the experimental protocol, LNA-injected mice were randomly assigned to either intravenous (i.v.) injection of 0.9% NaCl or 0.1 U/animal of insulin (Sigma). All mice were sacrificed by carbon dioxide (CO_2_) asphyxiation. All tissues were washed with 1× PBS, weighed, snap-frozen in liquid nitrogen, and stored at −80 °C.

### Enzyme-linked immunosorbent assay

Fibroblast Growth Factor 21 Mouse (FGF21) ELISA was obtained from Biovendor RD291108200R (Czech Republic), and performed using serum from CD and HFD-fed mice, Scr vs. *Mafg* LNA treated following the manufacturer’s instructions.

### Histological staining

For immunostaining, liver tissues were stored in PFA 4% for 24 h and then 70% ethanol for histological analysis. Transveral cryosections from paraffin-embedded liver were prepared, fixed, and stained by hematoxylin and eosin (H/E).

### RNA isolation and quantitative RT-PCR (qPCR) analysis

Total RNA was isolated from primary hepatocytes and whole tissues using peqGOLD TriFast (PEQLAB Biotechnologie). mRNA was reverse transcribed into complementary DNA using High Capacity cDNA reverse transcription kit (Applied Biosystems). Abundances of mRNAs were quantified by TaqMan Assay on Demand Kits (Applied Biosystems) according to the manufacturer’s protocol if not indicated otherwise. Abundances of lncRNAs were quantified using SYBR methodology using Select Master Mixes (Thermo Fisher). The relative abundance of mRNAs was calculated using comparative methods (2^−∆∆CT^) according to ABI Relative Quantification Methods. Transcript levels were normalized to hypoxanthine phosphoribosyltransferase 1 (*Hprt1*) or glyceraldehyde-3-phosphate dehydrogenase (*Gapdh*) expression; *Hprt* and *Gapdh* abundances remained unaffected across experimental conditions. SYBR primer sequences are provided in Supplementary Data 10.

### Reporting summary

Further information on research design is available in the Nature Research Reporting Summary linked to this article.

## Supplementary information

Supplementary Information

Description of Supplementary Data Files

Supplementary Data 1

Supplementary Data 2

Supplementary Data 3

Supplementary Data 4

Supplementary Data 5

Supplementary Data 6

Supplementary Data 7

Supplementary Data 8

Supplementary Data 9

Supplementary Data 10

Supplementary Data 11

Supplementary Data 12

Supplementary Data 13

Supplementary Data 14

Reporting Summary

## Data Availability

The source data underlying Fig. 1c–i, Fig. 2b, d, e, g, i, Fig. 3a–h, Fig. 4a–j, l–r, Fig. 5a–r, Fig. a–n, and Supplementary Fig. 1d–f, h–I, Fig. 2c–k, Fig. 3a–g, i–j, Fig. 4b, k–n, Fig. 5a–b, e are provided as Source Data file. Data from RNA-Seq are available under GEO Super Series GSE121346.

## Source Data

Source Data
