# Supplementary material for: A MAFG-lncRNA axis links systemic nutrient abundance to hepatic glucose metabolism
Source: Nat Commun. 2020 Jan 31;11:644. doi: 10.1038/s41467-020-14323-y (PMC6994702; doi:10.1038/s41467-020-14323-y)
Supplement: Supplementary file 1 — Supplementary Information [file 41467_2020_14323_MOESM1_ESM.pdf]

A MAFG-lncRNA axis links systemic nutrient abundance to hepatic glucose metabolism.

Pradas-Juni et al.

Supplementary Figure 1

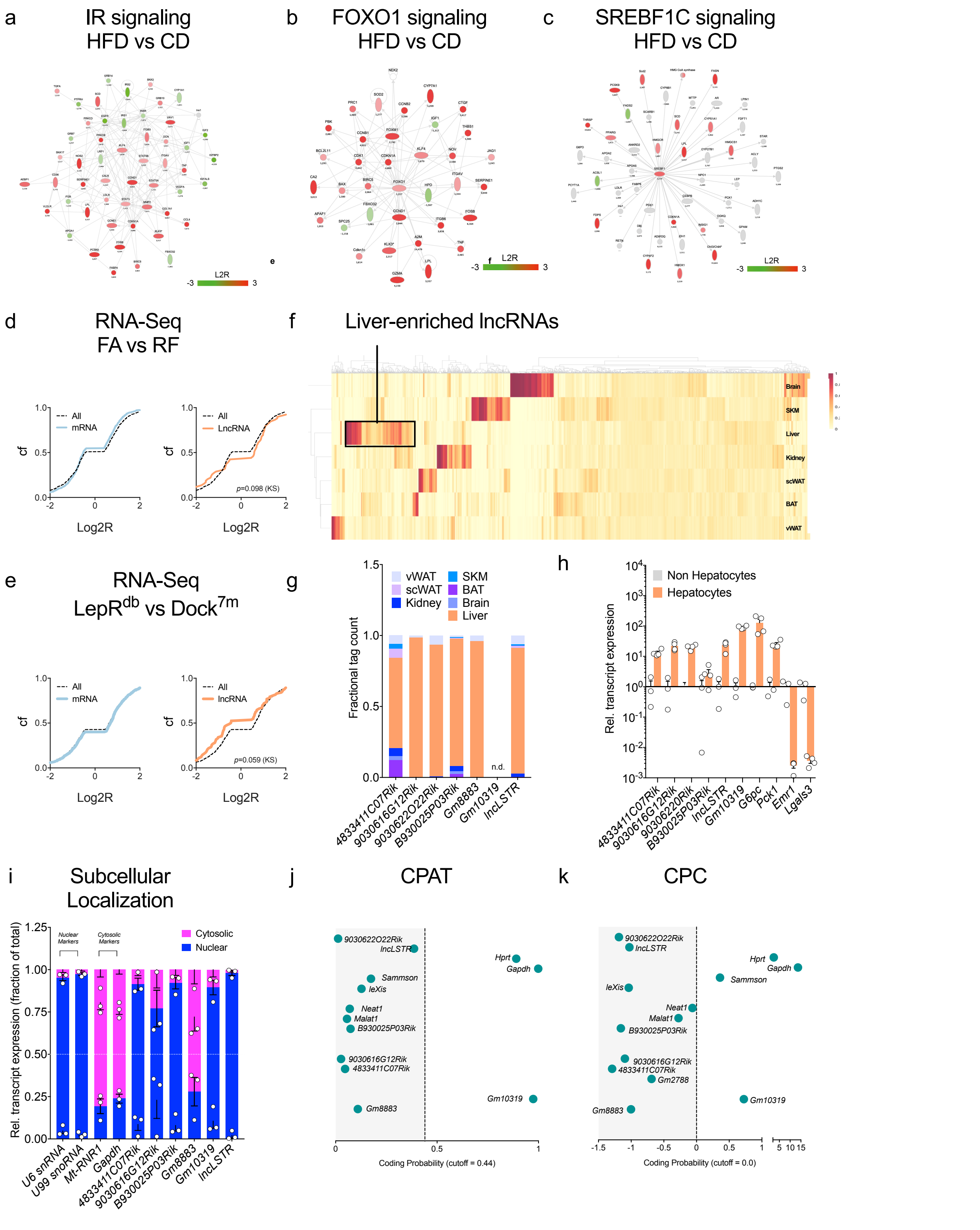

Supplementary Figure 1: Analysis of DIO signaling states and molecular characteristics of obesity lncRNAs

a-c Illustration of transcriptional activities of known obesity-associated signaling pathways like **a** IR **b** FOXO1 and **c** SREBP1C created using Ingenuity Pathway Analysis (IPA). Data for IPA network analysis are log2R-transformed expression changes from RNA-Seq performed in livers of C57BL/6N mice after 30 weeks of HFD- versus CD feeding (n=3 per group). **d** Log2R Cf of mRNA (blue) and lncRNA (orange) expression changes. Data are from RNA-Seq in liver of C57BL/6N mice after 16 h fasting (FA) vs 6 h refeeding (RF, n=4 each). Log2R Cf of hepatic mRNA (blue) and lncRNA (orange) expression changes in *LepR^db^* vs *Dock^7m^* mice (n=3 each). **d,e** Statistical differences between mRNAs and lncRNAs were assessed using Kolgomorov-Smirnov (KS) tests. *p*-values are given in the panels. **f** Heatmap of fractional read count in indicated tissues (SKM, skeletal muscle; scWAT, subcutaneous white adipose tissue; BAT, brown adipose tissue, vVAT, visceral adipose tissue). **g** Fractional read counts for seven liver-enriched obesity lncRNAs (L-DIO-lncRNAs). Data in **f,g** are derived from RNA-Seq performed in lean C57BL/6N mice (n=3 / tissue). **h** qPCR of indicated lncRNAs, hepatocyte-specific *G6pc* and *Pck1* and macrophage-specific *Emr1* and *Lgals3* expression from indicated cell fractions isolated of 8-weeks-old C57BL/6N mice (n=4). **i** qPCR for subcellular localisation of indicated lncRNAs and nuclear (*U6 snRNA*, *U99 snoRNA*), cytoplasmic and mitochondrial (*Mt-RNR1*, *Gapdh* mRNA) transcripts. Analysis were performed in 18- to 30-weeks-old C57BL/6N mice (n=3). **j,k** Coding potential prediction of indicated lncRNAs and mRNAs using **j** Coding-Potential Assessment Tool (CPAT) and **k** Coding Potential Calculator (CPC) transcript classifier tools. Source data are provided as a Source Data file.

Supplementary Figure 2

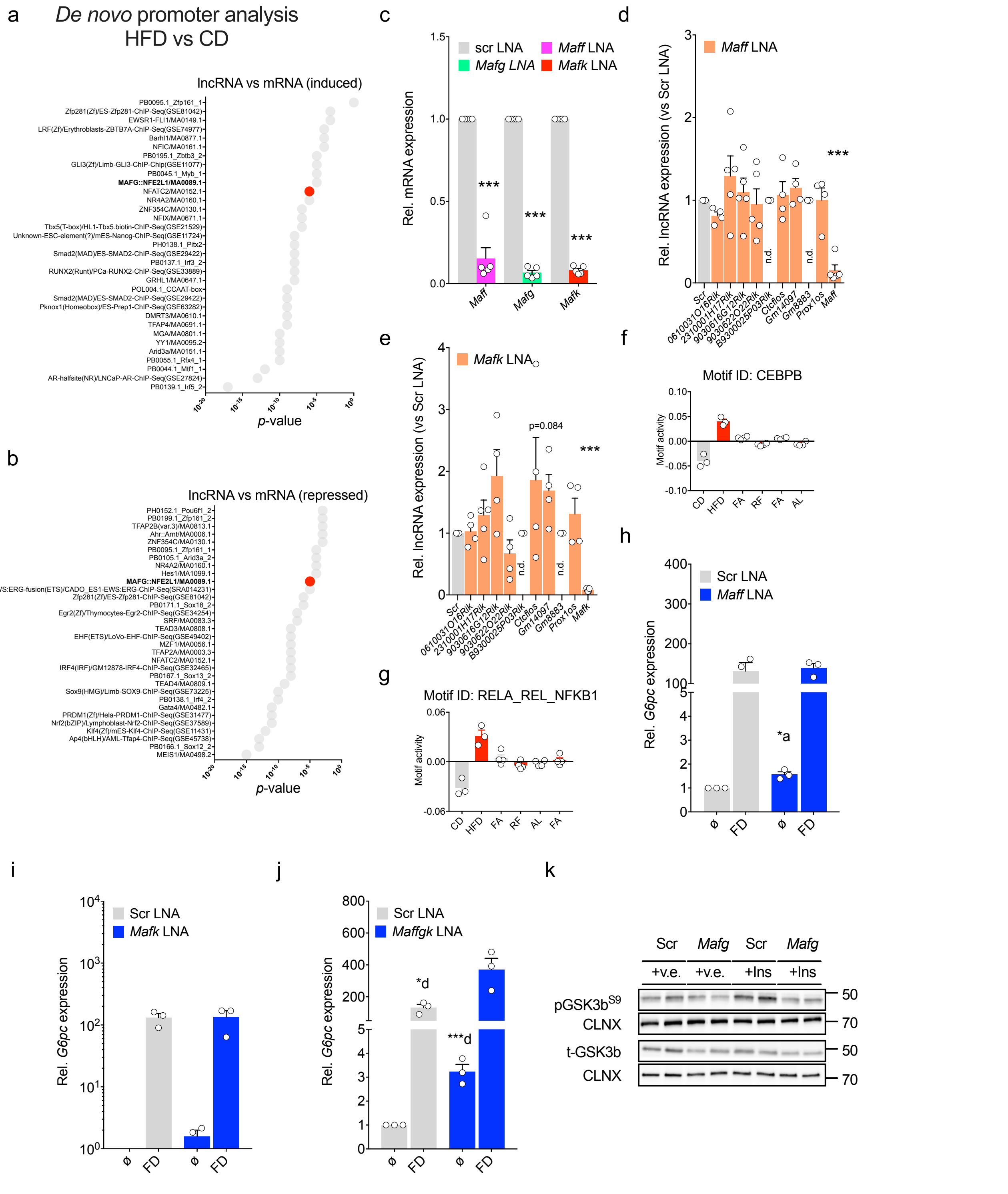

Supplementary Figure 2: De novo promoter analyses, smallMaf RNA interference and Mafg-dependent signaling states

a,b Differential promoter (-800/+100bp TSS) transcription factor binding site (TFBS) enrichment in indicated gene sets. The list of *de novo* motifs is represented as ordered motif enrichment *p*-values over background and MAFG:NFE2L1 TFBS highlighted in red. **c** qPCR of indicated *smMAF* mRNAs after transfection of primary hepatocytes with 100 nM of indicated *smMAF* isoform-specific or Scr LNA. Data represent n=5 independent experiments, each performed in triplicates. **d,e** qPCR of indicated lncRNAs after transfection of primary hepatocytes with 100 nM **e** *Maff* **f** *Mafk* or Scr LNAs. Data represent n=4 independent experiments, each performed in triplicates. n.d. = not done. **f,g** Motif activity for **f** CEBPB and **g** RELA-REL-NFKB1 TFBS across nutritional states and HFD-feeding analyzed using ISMARA[41] (n=3-4 per group). **h-j** qPCR for gluconeogenic *G6pc* expression after transfection of primary hepatocytes with **i** 100 nM *Maff* **j** *Mafk* or **k** combined *Maff*/*Mafg*/*Mafk* LNAs under basal conditions or stimulated with 10 µm Fsk plus 100 nM Dex (FD). Data represent n=3 independent experiments, each performed in triplicates. **k** Immunoblots of total GSK (t-GSK3b) and serine 9 phosphorylated GSK3B glycogen synthase kinase 3 beta (pGSK3b^{S9}) after transfection of primary hepatocytes with 100 nM *Mafg* or Scr LNAs. Cells were stimulated for 10 min with vehicle (*v.e.*) or 100 nM insulin. Separate membranes were loaded with equal amounts of lysate for total and phospho-isoform immunoblotting and equal total protein levels confirmed by CLNX for every membrane. Each blot represents n=3 immunoblots, with each immunoblot performed after an independent transfection, every transfection performed in separate hepatocyte isolation, and every preparation from n=2 mice. Bar graphs represent mean ± s.e.m. with all data points plotted and statistical differences were calculated using **c** unpaired or **d,e** paired, two-tailed Student's t-test or **h-j** one-way ANOVA with Bonferroni *post* correction for multiple testing. Superscripts depict group comparisons for *post* analysis ($^{a-f}$ = comparison vs column 1-6). **p*<0.05, ***p*<0.01, ****p*<0.001. Source data are provided as a Source Data file.

Supplementary Figure 3

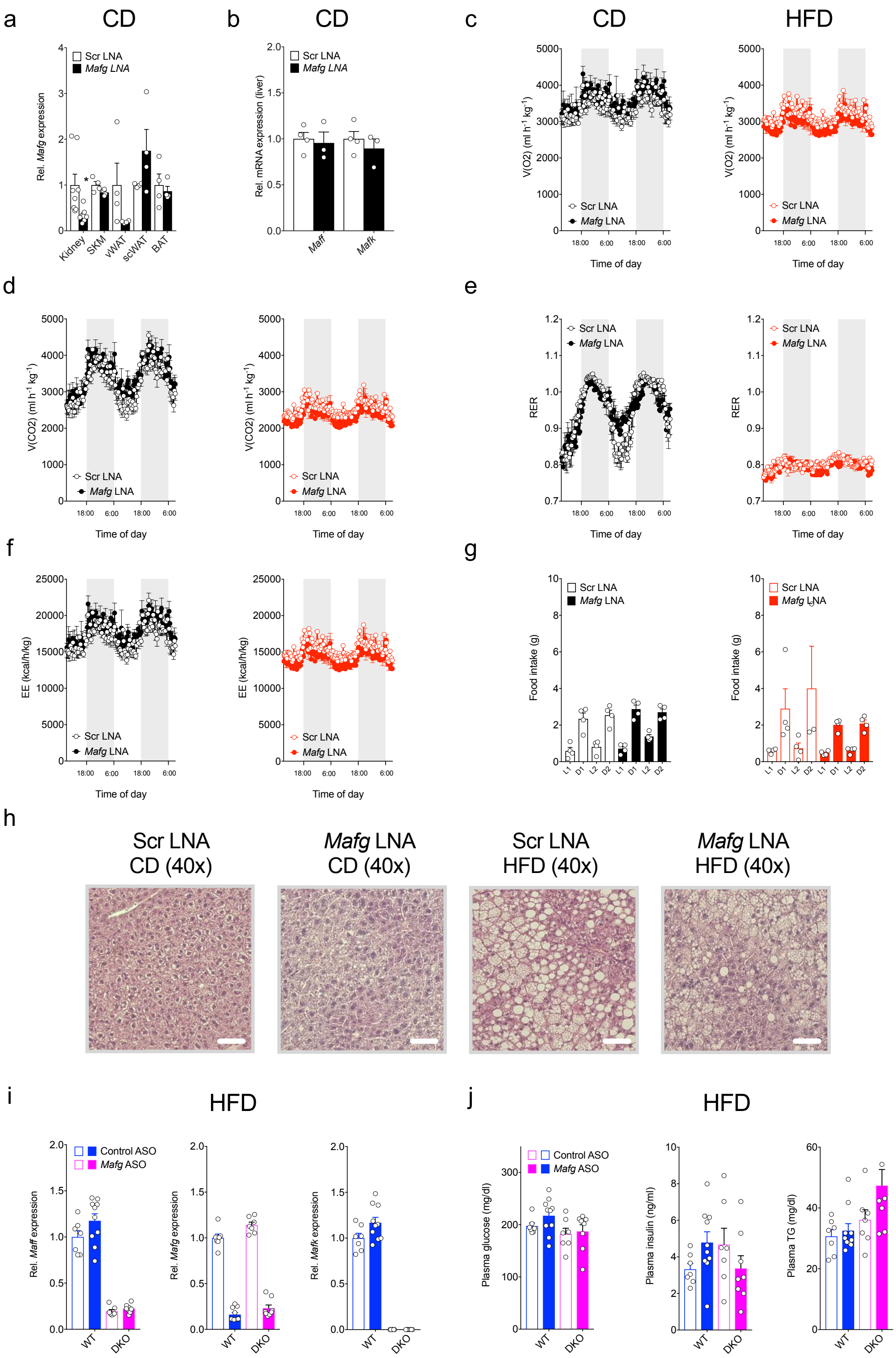

Supplementary Figure 3: Additional characteristics of Mafg loss- and gain-of-function mouse models:
a,b qPCR of **(a)** *Mafg* expression n indicated tissues from CD-fed mice at 21-weeks-of-age (n=8 for kidney, n=4 for SKM, vWAT, scWAT and BAT) and **b** *Maff* and *Mafk* mRNA in liver (n=4 for Scr LNA, n=3 for *Mafg* LNA) after 12 weeks of 10 mg kg^{-1} of anti-*Mafg* or Scr LNA treatment. **c-g** Indirect calorimetric determination of **c** oxygen (O2) consumption **d** carbon dioxide (CO2) production **e** RER assessment of substrate mobilisation **f** energy expenditure (EE) and **g** Food intake recordings during light (L) or dark (D) phases within two days (D1, D2) conducted in 16-weeks-old C57BL/6N mice fed 9 weeks of CD (black) or HFD (red) after 10 weeks of 10 mg kg^{-1} of *Mafg* or Scr LNA treatment (n=4 per genotype and diet). **h** Representative photomicrograph of haematoxylin / eosin (HE) staining in livers from CD- or HFD-fed C57BL/6N mice after 12 weeks of *Mafg* or Scr LNA treatment. **i** qPCR of *Maff, Mafg* and *Mafk* expression in liver of HFD-fed C57BL/6N or *Maff/Mafk*$^{-/-}$ (DKO) after 20 weeks of 5 mg kg^{-1} *Mafg* or Control oligonucleotide (ASO) treatment (n=7 for Control ASO in C57BL/6, n=7 for Control ASO in DKO, n=10 for *Mafk* ASO in C57BL/6N and n=8 for *Mafg* ASO in DKO mice). **j** Blood glucose, insulin and triglyceride (TG) levels in HFD-fed C57BL/6N or DKO mice at 16-weeks-of-age after 10 weeks of 5 mg kg^{-1} of *Mafg* or Control ASO treatment (n=13 for *Mafg* ASO (n=7 for C57BL/6, n=6 for DKO background) and n=18 for Control ASO (n=10 for C57BL/6, n=8 for DKO background). **h** Scale bars in images represent 100 µm. Bar graphs represent mean ± s.e.m. with all data. **p*<0.05, ***p*<0.01, ****p*<0.001. Source data are provided as a Source Data file.

Supplementary Figure 4

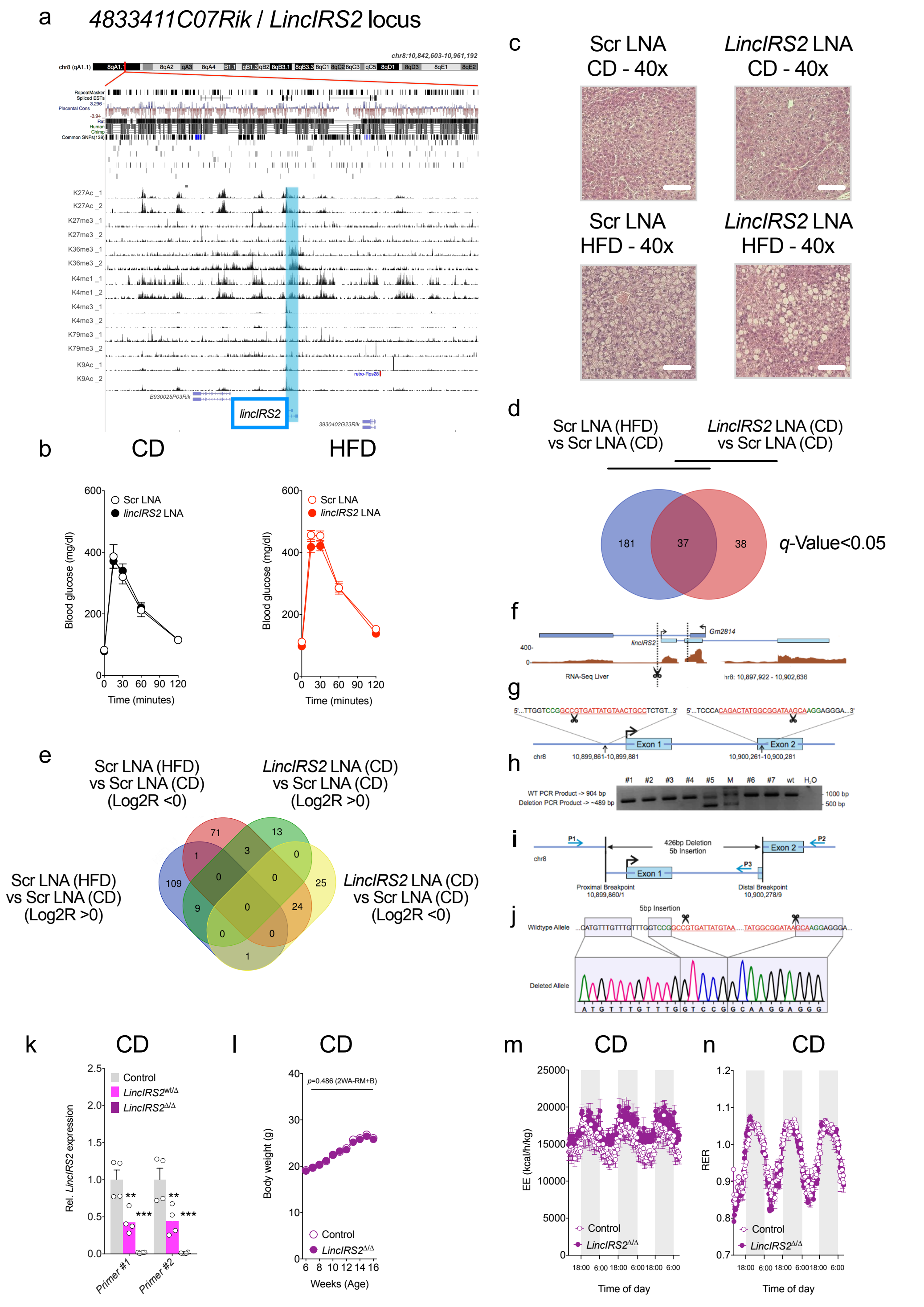

Supplementary Figure 4: LincIRS2 chromatin states, generation and characteristics of $LincIRS2^{\Delta/\Delta}$ mouse line.

a UCSC Browser of histone H3 modifications determined by ChiP-Seq. Data were from NIH Roadmap Epigenomics Project[71]. **b** GTT in 12-weeks-old C57BL/6N mice fed 5 weeks of CD *(left)* or HFD *(right)* after 6 weeks of 10 mg kg^{-1} of *LincIRS2* or Scr LNA treatment (n=8 each). **c** Representative photomicrograph of livers resected from Scr LNA and *LincIRS2* LNA-treated mice. **d,e** Venn diagrams depicting overlaps between genes differentially expressed in livers of CD- and HFD- fed C57BL/6N mice after 10 weeks of 10 mg kg^{-1} of *LincIRS2* or Scr LNA treatment (n=4 each). **f** Details of CRISPR/Cas9-mediated *4833411C07Rik*/*LincIRS2* knockout. Dotted lines show sites targeted by guide RNA (gRNA)-mediated CRISPR/Cas9 activity. **g** Representation of the engineered *LincIRS2* locus. Sequences of the crRNAs recruiting Cas9 are shown in red. **h** Agarose gel electrophoresis of PCR products amplified on genomic DNA isolated from 7 individual founder mice obtained from pronuclear injection of CRISPR/Cas9 ribonucleoprotein complexes; *M*, Marker, *wt*, C57BL/6N wildtype; H2O, no template control; 904bp, wildtype PCR product; 500bp size of deletion PCR products. **i** Scheme depicting the $LincIRS2^{\Delta}$ allele with proximal and distal breakpoints. P1, P2 and P3 indicate PCR primers used for genotyping and sequencing. **j** Chromatogram showing DNA sequence of the $LincIRS2^{\Delta}$ in comparison to wildtype alleles. **k** qPCR of *LincIRS2* expression in livers from indicated genotypes (n=4 each). **l** Body weight in lean $LincIRS2^{\Delta/\Delta}$ and Control mice (n=8 each). **m,n** Indirect calorimetric determination of **m** EE and **n** RER-mediated assessment of substrate mobilisation in 16-weeks-old $LincIRS2^{\Delta/\Delta}$ and Control mice (n=8). **c** Scale bars in images represent 100 µm. Bar graphs represent mean ± s.e.m. with all data points plotted and statistical differences were calculated using **k** unpaired, two-tailed Student's t-test or **l** two-way ANOVA with Bonferroni *post* correction for multiple testing $^{**}p<0.01$. Source data are provided as a Source Data file.

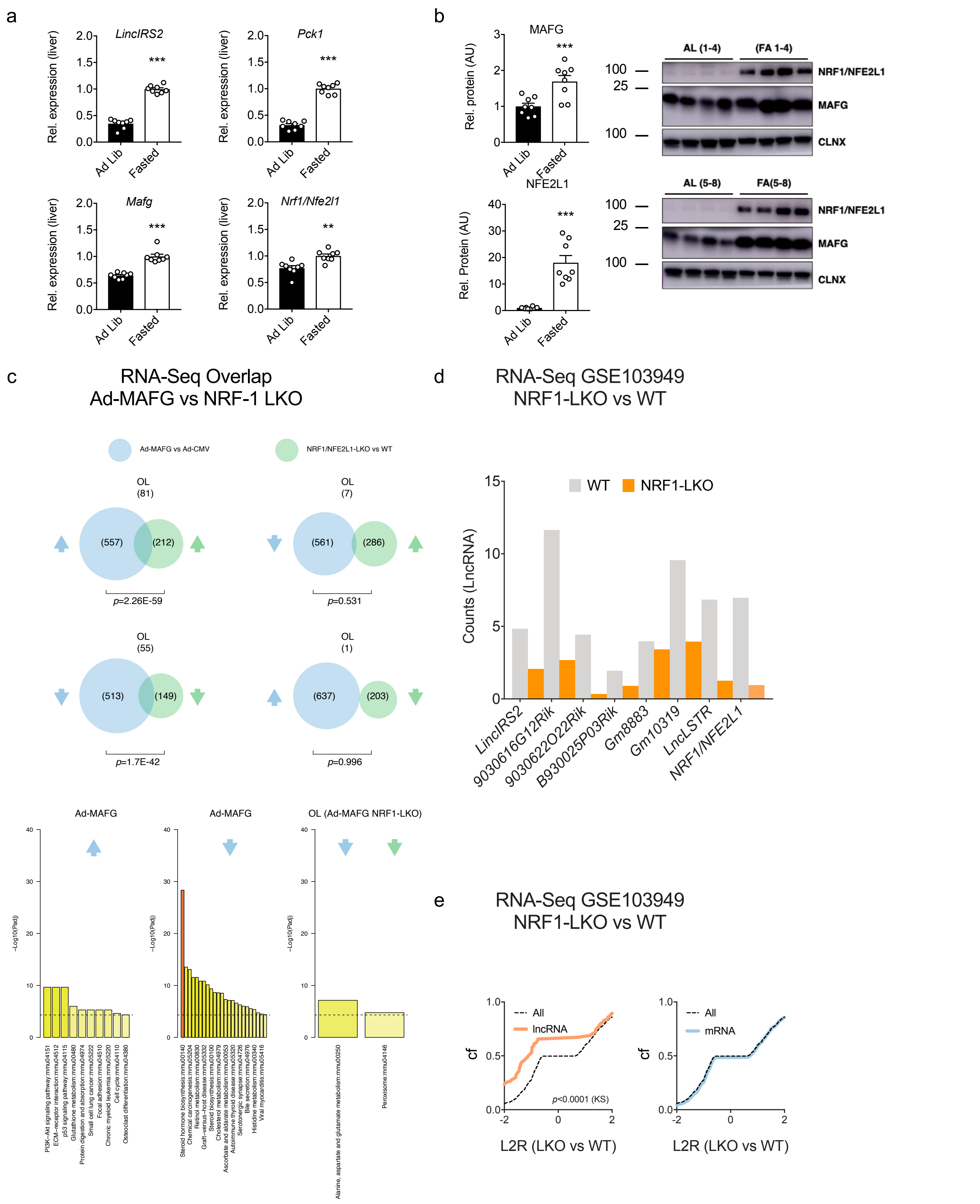

Supplementary Figure 5: Transcriptomic analysis of fasting-induced changes in NRF1/NFE2L1 and MAFG signaling.
a PCR for indicated mRNAs and *LincIRS2* in C57BL/6N and **b** immunoblot for MAFG and NRF1/NFE2L1 after *Ad libitum* feeding (Ad Lib) vs 16 h fasting (n=8 each). **c** Venn diagram illustrating DGE overlap and enriched GO categories after RNA-Seq analysis of mice injected with Ad-MAFG and animals conditionally null for NRF1/NFE2L1 in liver (NRF-1 LKO). **d** Expression of indicated L-DIO-lncRNAs as determined by RNA-Seq of NRF-1 LKO mice. Ensembl transcript ID and statistical information is provided below the graph. **e** Re-analysis of public RNA-Seq (GEO ID: 103949) and Log2R Cf of hepatic mRNA (blue) and lncRNA (orange) hepatic expression changes in mice conditionally null for *Nrf1/Nfe2l1* in hepatocytes. **e** Statistical differences between mRNAs and lncRNAs were assessed using Kolgomorov-Smirnov (KS) tests. *p*-values are given in the panels. Bar graphs represent mean ± s.e.m. with all data points plotted and statistical differences were calculated using **a,b** unpaired, two-tailed Student's t-test. **p*<0.05, ***p*<0.01, ****p*<0.001. Source Data are provided as a Source Data file.
