# Supplementary material for: A MAFG-lncRNA axis links systemic nutrient abundance to hepatic glucose metabolism
Source: Nat Commun. 2020 Jan 31;11:644. doi: 10.1038/s41467-020-14323-y (PMC6994702; doi:10.1038/s41467-020-14323-y)
Supplement: Supplementary file 2 — Description of Supplementary Data Files [file 41467_2020_14323_MOESM2_ESM.docx]

**Description of Additional Supplementary Files:**

1. **File Name: F1_Supplementary Data 1_RNA-SEQ_(HFD--CD).xlsx**

**Description: Analysis of Analysis of RNA-Sequencing of livers from Control Diet (CD) or High-fat-diet (HFD)-fed C57BL/6N mice.**

1. **File Name: F1_Supplementary Data 2_RNA-SEQ_(AL--FA).xlsx**

**Description: Analysis of RNA-Sequencing of livers from Ad Libitum (AL) or overnight fasted (FA) C57BL/6N mice.**

1. **File Name: F1_Supplementary Data 3_RNA-SEQ_(FA--RF).xlsx**

**Description: Analysis of RNA-Sequencing of livers from overnight fasted (FA) or 6 h re-fed (RF) C57BL/6N mice.**

1. **File Name: F1_Supplementary Data 4_RNA-SEQ_(Dock7M--DBDB).xlsx**

**Description: Analysis of RNA-Sequencing of livers from Leprdb (DBDB) or Dock7m (misty) mice.**

1. **File Name: F1_Supplementary Data 5_RNA-SEQ_(HS_T2D--OB--LEAN).xlsx**

**Description: Analysis of RNA-Sequencing from human livers biopsies of Lean, Obese (øT2D) and Obese (+T2D) patients.**

1. **File Name: F1_Supplementary Data 6_RNA-SEQ_(Ad-MAFG--Ad-CMV).xlsx**

**Description: Analysis of RNA-Sequencing of livers from C57BL/6 mice injected with Ad-MAFG or Ad-CMV.**

1. **File Name: F1_Supplementary Data 7_RNA-SEQ_(SCR_LNA_CD--SCR_LNA_HFD).xlsx**

**Description: Analysis of RNA-Sequencing of livers from CD- or HFD-fed C57BL/6N mice injected with Scr LNA.**

1. **File Name: F1_Supplementary Data 8_RNA-SEQ_MAFG_LNA_HFD--SCR_LNA_HFD.xlsx**

**Description: Analysis of RNA-Sequencing of livers from HFD-fed C57BL/6N mice injected with Scr LNA or Mafg LNA.**

1. **File Name: F1_Supplementary Data 9_RNA-SEQ_(SCR_LNA_CD--LNCIRS2_LNA_CD).xlsx**

**Description: Analysis of RNA-Sequencing of livers from CD- or HFD-fed C57BL/6N mice injected with Scr LNA.**

1. **File Name: F1_Supplementary Data 10_Oligonucleotide_Sequences.xlsx**

**Description: Oligonucleotide sequences for SYBR qPCR analysis, LNA GapmeR sequences, ASO sequences, TaqMan qPCR assays and sgRNA sequences for CRISPR/Cas9 genome editing used in this study.**

1. **File Name: F1_Supplementary Data 11_RNA-SEQ_NRF1_NFE2L1-LKO.xlsx**

**Description: Analysis of RNA-Sequencing of livers from liver-specific NFE2L1/NRF knockout versus wildtype mice (CD feeding).**

1. **File Name: F1_Supplementary Data 12_Comparisions_Gene-Overlap.xlsx**

**Description: Biotype-level gene overlap between groups as determined by analysis of RNA-Sequencing.**

1. **File Name: F4_Pradas_Juni_Supplementary Data 13.pdf**

**Description: Gene models of syntenic protein-coding genes and lncRNAs in the indicated regions.**

1. **File Name: F4_Pradas_Juni_Supplementary Data 14.pdf**

**Description: Sanger-Sequencing of the five most likely Cas9 off-target for each sgRNA.**
