# Supplementary material for: A MAFG-lncRNA axis links systemic nutrient abundance to hepatic glucose metabolism
Source: Nat Commun. 2020 Jan 31;11:644. doi: 10.1038/s41467-020-14323-y (PMC6994702; doi:10.1038/s41467-020-14323-y)
Supplement: Supplementary file 15 — Supplementary Data 13 [file 41467_2020_14323_MOESM15_ESM.pdf]

Supplementary Data 13

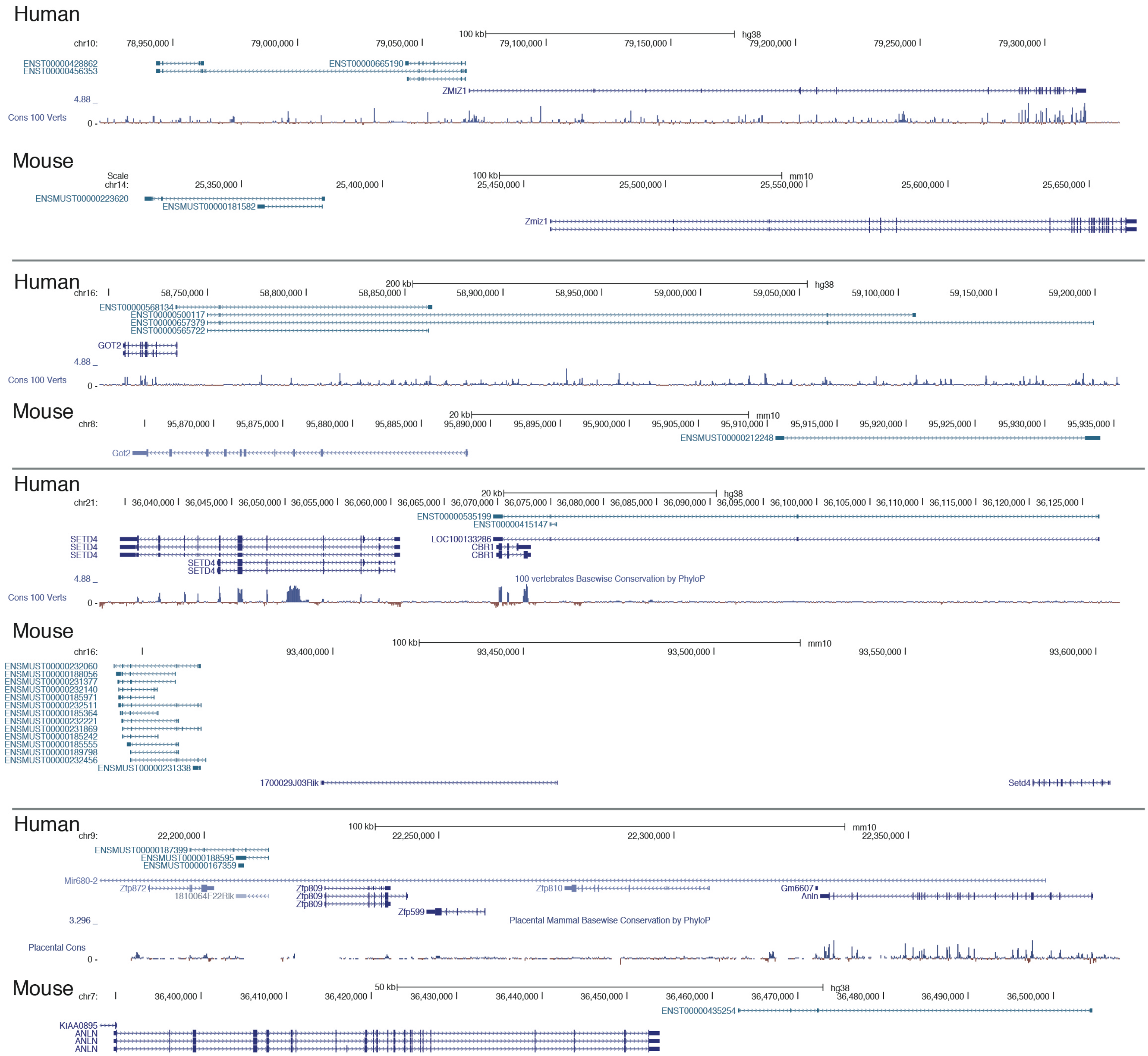

Gene models of protein-coding genes and lncRNAs in the indicated regions are shown in the indicated species. Conservation scores and protein-coding gene models are taken from the UCSC genome browser.
