# Supplementary material for: A MAFG-lncRNA axis links systemic nutrient abundance to hepatic glucose metabolism
Source: Nat Commun. 2020 Jan 31;11:644. doi: 10.1038/s41467-020-14323-y (PMC6994702; doi:10.1038/s41467-020-14323-y)
Supplement: Supplementary file 16 — Supplementary Data 14 [file 41467_2020_14323_MOESM16_ESM.pdf]

Pradas-Juni et al. Supplementary Data 14

For each off-target candidate, the result figures are organized as follows:

(A) Sanger sequences of each off-target PCR amplicon are showed, including both forward (Fw) and reverse (Rv) primers used for the PCR reaction as well as the alternative PAM region.

(B) The alternative PAM region (highlighted in light blue) is visualized within the Sanger PCR amplicon at the Genome Browser site and DNA sequence trace chromatogram confirms no mismatches or rearrangements.

(C) The comparison between the control sequence ("Genomic") and the Sanger-seq indicates 100% identity between the two sequences.

Sanger Seq
>#1_3prime_intron_Ivd_Sanger-Seq
GGTGTGACCCAGAGAGACTCCGCCTCTAACCAAAGTGACAGAGGTAACTAAGATTAACATCAGTGT
TCTGTGCTCTGCCCCCAGGAGCATGCGGATAAGCAGAGACCAAAGCGGATAAGCAGGGACCAAAG
AAGGTAGCCAGAGAGGCTGGGTCAAGTTTGAAGACAGTAAAACTGTCCACAGAGTTGAGGAAGAG
GAAAATGGAAAAGAAGCCAGGAGCAGGA
>Fw
TGGCAACAAGTTCTGGATCACCA
>Rv
CACCTGTGTGGCTGGATTTGT
>PAM_region_chr2:118,873,418-118,873,440
GAGACCAAAGCGGATAAGCAGGG

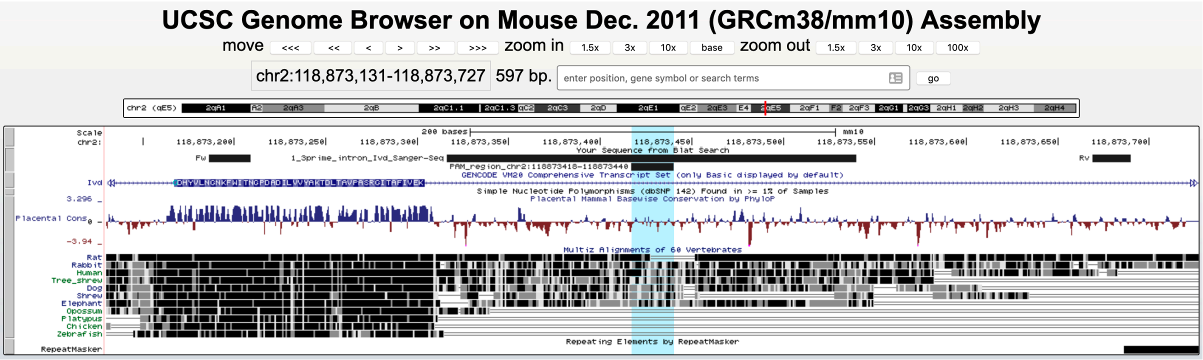

PAM region

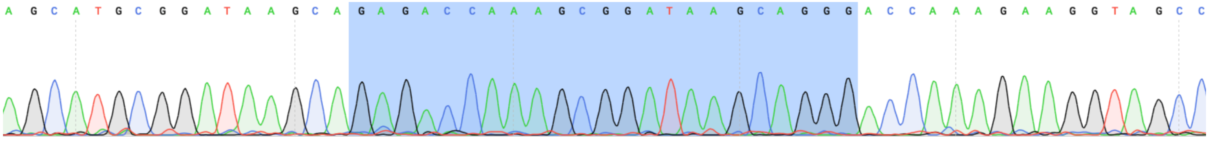

Genomic chr2 :

tgatgctgat atcctagtcg tgtatgccaa gacagatttg accgctgtgc 118873266
cagcttctcg gggcatcaca gccttcattg tggagaaggt gagggtggtg 118873316
GGTGTGACCC AGAGAGACTC CGCCTCTAAC CAAAGTGACA GAGGTAACTA 118873366
AGATTAACAT CAGTGTTCTG TGCTCTGCCC CCAGGAGCAT GCGGATAAGC 118873416
AGAGACCAAA GCGGATAAGC AGGGACCAAA GAAGGTAGCC AGAGAGGCTG 118873466
GGTCAAGTTT GAAGACAGTA AAACTGTCCA CAGAGTTGAG GAAGAGGAAA 118873516
ATGGAAAAGA AGCCAGGAGC AGGAactgca ggggaggctg aggcatgggt 118873566
gggaatgtga atgagacctg caaacctctg atagtaagga caagacaaga 118873616
cacatcagga gttaaaactc gagg

cDNA 1_3prime_intron_Ivd_Sanger-Seq

GGTGTGACCC AGAGAGACTC CGCCTCTAAC CAAAGTGACA GAGGTAACTA 50
AGATTAACAT CAGTGTTCTG TGCTCTGCCC CCAGGAGCAT GCGGATAAGC 100
AGAGACCAAA GCGGATAAGC AGGGACCAAA GAAGGTAGCC AGAGAGGCTG 150
GGTCAAGTTT GAAGACAGTA AAACTGTCCA CAGAGTTGAG GAAGAGGAAA 200
ATGGAAAAGA AGCCAGGAGC AGGA

Sanger Seq
>#2_3prime_intron_Ikzf4_Sanger-Seq
CCTTTTACCCTGGAAAGGTATATGACTTGAAACCAGAACTTTGGTAGAAGACCTTCCAAAAGACCAT
TGCCCAAAATAGCTCAGGGAGTCCTGTTTGACCCAAGACTGAGGAGGATAAGCAGGGGCGCTGTAC
TCCCCAATACCCTCCATTTACATTTTGAGATAAGGCCCCAGAATTCTGTCCCTTTTTGACTAAAAATCT
CTCCCCACTTCTGCAAACCTTT
>Fw
TGCAGGTGAAAGATCAGG
>Rv
TGGCGCTTCAGTGTAGAT
>PAM_region_chr10:128,640,547-128,640,569
AAGACTGAGGAGGATAAGCAGGG

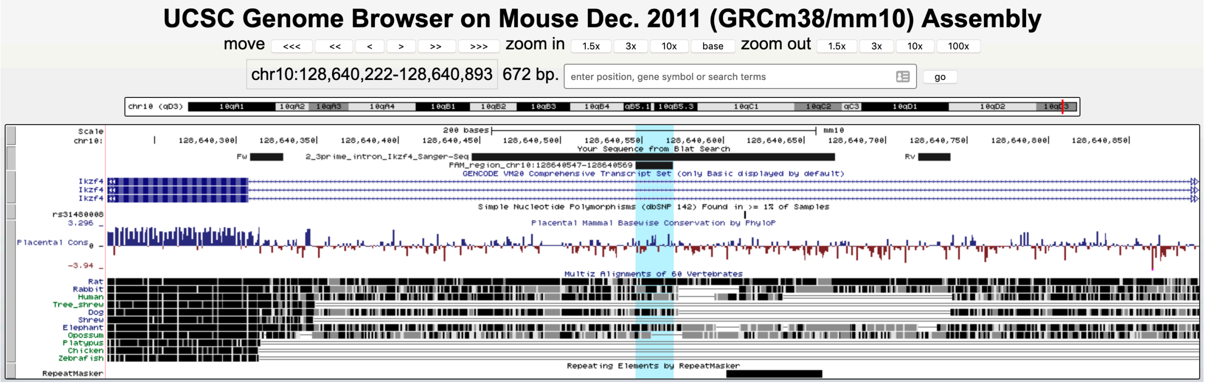

PAM region

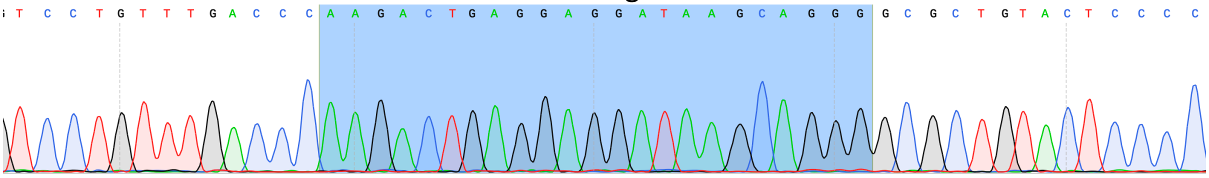

Genomic chr10 :

```
aggggtgtgg aagagcggag ggcaagcttt ctgagcgcac tctggagggg  128640395
cagtgggtgc tacacatacc aggcctatgt tatagaggtc acctgggggc  128640445
CCTTTTACCC TGGAAAGGTA TATGACTTGA AACCAGAACT TTGGTAGAAG  128640495
ACCTTCCAAA AGACCATTGC CCAAAATAGC TCAGGGAGTC CTGTTTGACC  128640545
CAAGACTGAG GAGGATAAGC AGGGGCGCTG TACTCCCCAA TACCCTCCAT  128640595
TTACATTTTG AGATAAGGCC CCAGAATTCT GTCCCTTTTT GACTAAAAAT  128640645
CTCTCCCCAC TTCTGCAAAC CTTTtttggt gttgagatcc accacaaggc  128640695
cttacacgtg gacacacccc accgccgatc tacactgaag cgccaagaat  128640745
ccttccagag ggcccagtac ccgt
```

cDNA 2_3prime_intron_Ikzf4_Sanger-Seq

```
CCTTTTACCC TGGAAAGGTA TATGACTTGA AACCAGAACT TTGGTAGAAG  50
ACCTTCCAAA AGACCATTGC CCAAAATAGC TCAGGGAGTC CTGTTTGACC  100
CAAGACTGAG GAGGATAAGC AGGGGCGCTG TACTCCCCAA TACCCTCCAT  150
TTACATTTTG AGATAAGGCC CCAGAATTCT GTCCCTTTTT GACTAAAAAT  200
CTCTCCCCAC TTCTGCAAAC CTTT
```

Sanger Seq
>#3_3prime_exon_Cyp4a14_Sanger-Seq
AGACCACATAGGGGCATAAGTGATCAGAAATATAAAATATCAATATGTTCCAACCCTCCATCCTTTCT
CTTCAACACTTGCCCACCATTACCTTGATCCTCCTGAGAATGGCAGATAAGCATGGCTATGGTGAGA
AGAATCTGGTGCAAATCTAGAGGGGTCAAACACCTGCAGAGAGAGCACAGGGCCAGGATGGATGG
TGTCAAGCCCATTTCCAATGCAAA
>Fw
CCTTTTGGCACAAAACAC
>Rv
CACAGCCACAATTTCCAT
>PAM_region_chr4:115,489,799-115,489,821
CTGAGAATGGCAGATAAGCATGG

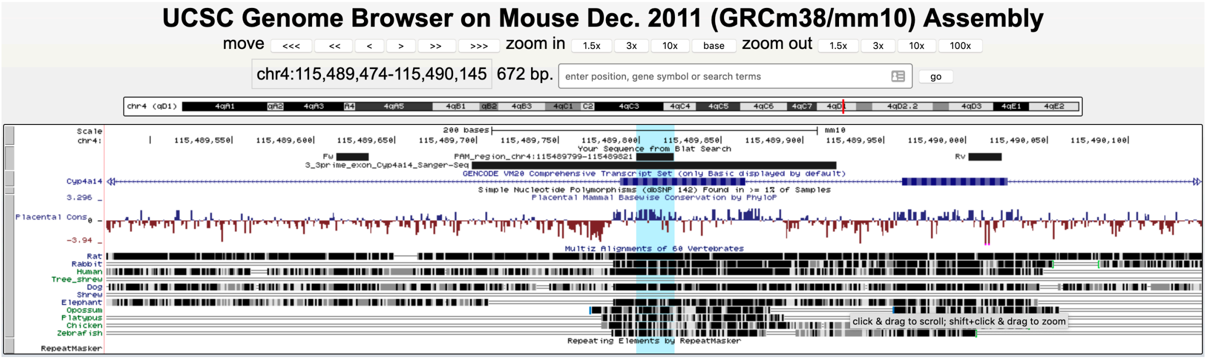

PAM region

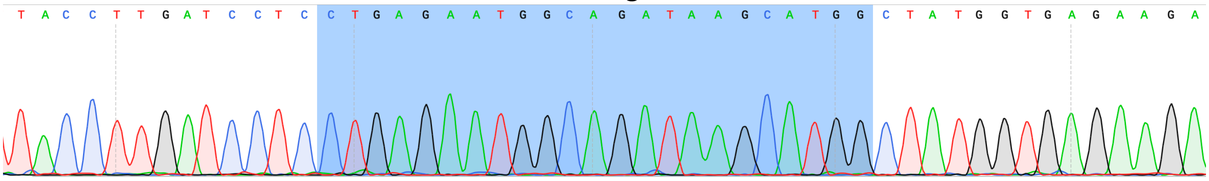

Genomic chr4 :

```
gaattgctgc tgtctttcct tttggcacaa aacacatata catcatacaa  115489647
tccccccccc acacacacac acacatagac caacagaaat acatgcaaat  115489697
AGACCACATA GGGGCATAAG TGATCAGAAA TATAAAATAT CAATATGTTC  115489747
CAACCCTCCA TCCTTTCTCT TCAACACTTG CCCACCATTA CCTTGATCCT  115489797
CCTGAGAATG GCAGATAAGC ATGGCTATGG TGAGAAGAAT CTGGTGCAAA  115489847
TCTAGAGGGG TCAAACACCT GCAGAGAGAG CACAGGGCCA GGATGGATGG  115489897
TGTCAAGCCC ATTTCCAATG CAAAagggta cagtatggtt acagtcttct  115489947
ccagatttcc ataccttggg gtttggccag aaacgtgggt tatgatgtag  115489997
gccataaatg gaaattgtgg ctgt
```

cDNA 3_3prime_exon_Cyp4a14_Sanger-Seq

```
AGACCACATA GGGGCATAAG TGATCAGAAA TATAAAATAT CAATATGTTC   50
CAACCCTCCA TCCTTTCTCT TCAACACTTG CCCACCATTA CCTTGATCCT  100
CCTGAGAATG GCAGATAAGC ATGGCTATGG TGAGAAGAAT CTGGTGCAAA  150
TCTAGAGGGG TCAAACACCT GCAGAGAGAG CACAGGGCCA GGATGGATGG  200
TGTCAAGCCC ATTTCCAATG CAAA
```

Sanger Seq

>#4_3prime_intergenic_Gm25335-Magi2_Sanger-Seq

TTTTGGTGATAATTAATAGGAGAAAGGAAATTAAAAGGTTAAACTATCTTAGCTTGCCACAATGCAA
ACACTTAAAGATAAAATATCTGTAGCTAAAAGATACTCTGGTAAACAAACTATGAAGGATAATCAAG
GATGAAGAAAACCTGGAAGAAGATAAAATACTTCCAAAATATAAGATCATTTTTAGTATTGTAATTT
GTAGCAAGTTATTTAATTAA

>Fw

GTGAGGCTCTGGACTGAGTACTGT

>Rv

AGTCCAGCATCATTTTGCCCCC

>PAM_region_chr5:19,674,063-19,674,085

CAAACTATGAAGGATAATCAAGG

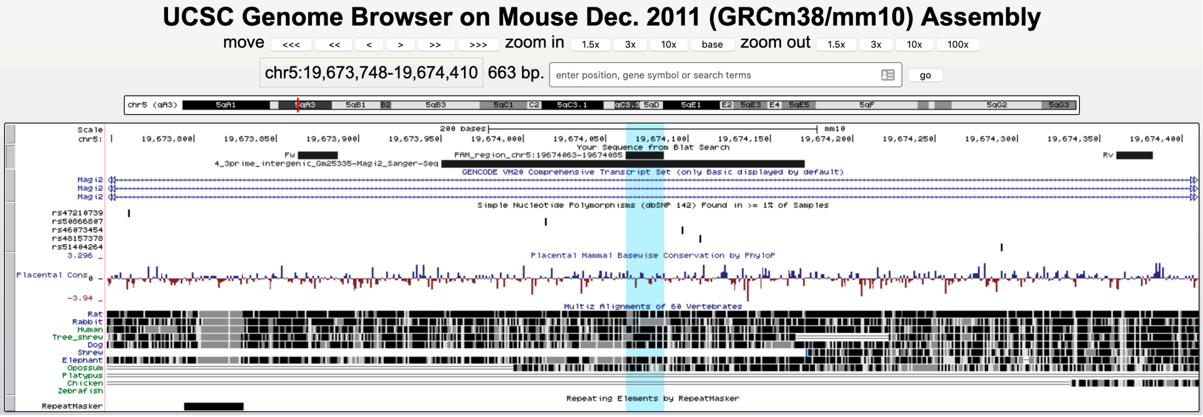

PAM region

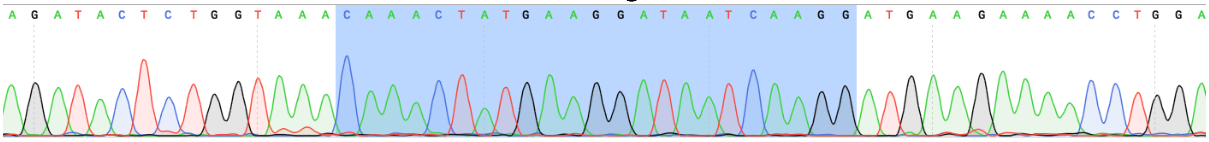

Genomic chr5 :

```
gacagctaga aaagtgaggc tctggactga gtactgtttt agatctggag  19673900
tgaatgtccc atgactgagc atatttatgt acaaaactaa ataaaaatta  19673950
TTTTGGTGAT AATTAATAGG AGAAAGGAAA TTAAAAGGTT AAACTATCTT  19674000
AGCTTGCCAC AATGCAAACA CTTAAAGATA AAATATCTGT AGCTAAAAGA  19674050
TACTCTGGTA AACAAACTAT GAAGGATAAT CAAGGATGAA GAAAACCTGG  19674100
AAGAAGATAA AATACTTCCA AAATATAAGA TCATTTTTAG TATTGTAATT  19674150
TGTAGCAAGT TATTTAATTA Atgaaggaaa tttcacactt attcttaaaa  19674200
gcttcttcaa gttcttcctt ggacggaaaa tacaactagc cactgaaaca  19674250
ggttgctaaa catttggtaa t
```

cDNA 4_3prime_intergenic_Gm25335-Magi2_Sanger-Seq

```
TTTTGGTGAT AATTAATAGG AGAAAGGAAA TTAAAAGGTT AAACTATCTT  50
AGCTTGCCAC AATGCAAACA CTTAAAGATA AAATATCTGT AGCTAAAAGA  100
TACTCTGGTA AACAAACTAT GAAGGATAAT CAAGGATGAA GAAAACCTGG  150
AAGAAGATAA AATACTTCCA AAATATAAGA TCATTTTTAG TATTGTAATT  200
TGTAGCAAGT TATTTAATTA A
```

Sanger Seq
>#5_3prime_exon_Plxna3_Sanger-Seq
CCCATTGATAAGCCTCCTAGCTTCCGAGGTGAGGGGTGACATGGGGCCAAGGAGCATGGCTCCTGG
GGCAGGATTGGCACTGCTGGGGGCAAGTCTGTAGGTCCTAGAGCCCCCTGCTTTTCTGCCATTGCCT
GCTCCATCAGTAGTCTCCTGTGCCTGCAGCCCTTCTTTACAAGTGCTGGGCTCAGCGGCCTAGCTGTG
GCCTCTGCCTCAAGGCTGATCCCC
>Fw
AGGGATTCCTAAAGTGGGCAAGGA
>Rv
TCGGGCACCCTTCTGGCTTG
>PAM_region_chrX:74,335,950-74,335,972
CCCTGCTTTTCTGCCATTGCCTG

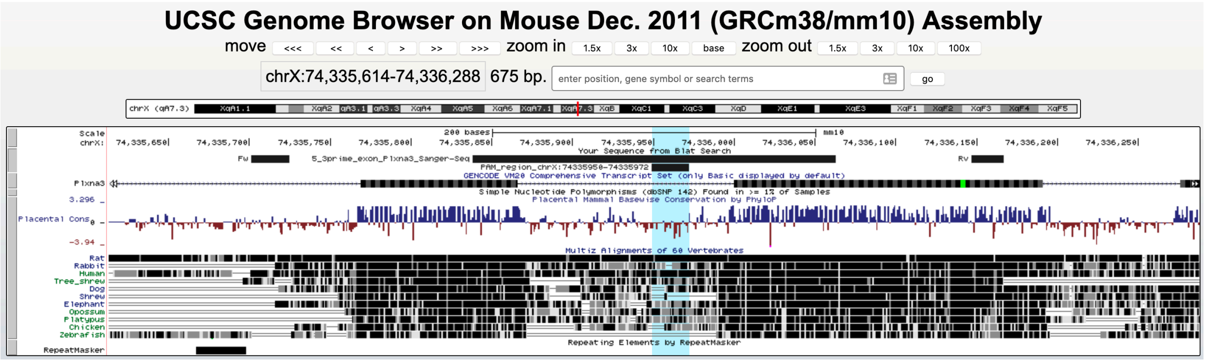

PAM region

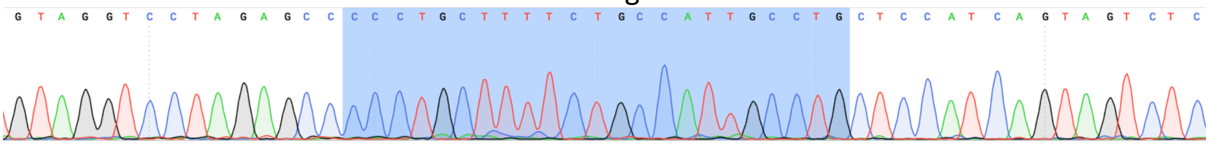

Genomic chrX :

```
atttgtgctc atgtggttcc atttttctta gtacttctat gaaggtgatg   74335788
agtttggtga caccgaactg gacttctctg tggtttggga tggagatttc   74335838
CCCATTGATA AGCCTCCTAG CTTCCGAGGT GAGGGGTGAC ATGGGGCCAA   74335888
GGAGCATGGC TCCTGGGGCA GGATTGGCAC TGCTGGGGGC AAGTCTGTAG   74335938
GTCCTAGAGC CCCCTGCTTT TCTGCCATTG CCTGCTCCAT CAGTAGTCTC   74335988
CTGTGCCTGC AGCCCTTCTT TACAAGTGCT GGGCTCAGCG GCCTAGCTGT   74336038
GGCCTCTGCC TCAAGGCTGA TCCCCgattc aactgtggct ggtgcatctc   74336088
agagcacagg tgccagctga gggctcactg cccagctccc aaaagtaact   74336138
ggatgcaccc aagccagaag ggtgc
```

cDNA 5_3prime_exon_Plxna3_Sanger-Seq

```
CCCATTGATA AGCCTCCTAG CTTCCGAGGT GAGGGGTGAC ATGGGGCCAA   50
GGAGCATGGC TCCTGGGGCA GGATTGGCAC TGCTGGGGGC AAGTCTGTAG   100
GTCCTAGAGC CCCCTGCTTT TCTGCCATTG CCTGCTCCAT CAGTAGTCTC   150
CTGTGCCTGC AGCCCTTCTT TACAAGTGCT GGGCTCAGCG GCCTAGCTGT   200
GGCCTCTGCC TCAAGGCTGA TCCCC
```

Sanger Seq
>#1_intergenic_Zfp428-Irgq
ACGCAGTAAGCTTAGCGTTGTACGGAGCACACAACCAACGTCATCATCACGCACCCCTGTACAGAAA
AGGACCTCTCCACTTGCCCTTGCGAAGACTTGCCTGCCGTGACTATATTAGTGCCGGACTGCAACCTT
GAACCTGACTCGGGGACAATGGTGGTGCAACAGATCACTGGAGTCTGGAGACTAGGAAAAACTATC
CCGCTCTCGTCCGGTGGCAGAG
>Fw
TCACGCTGCTCCCATGCACT
>Rv
ATGCGCGTGCGCAGAAAGTT
>PAM_region_chr7:24,530,430-24,530,452
GGCACTAATATAGTCACGGCAGG

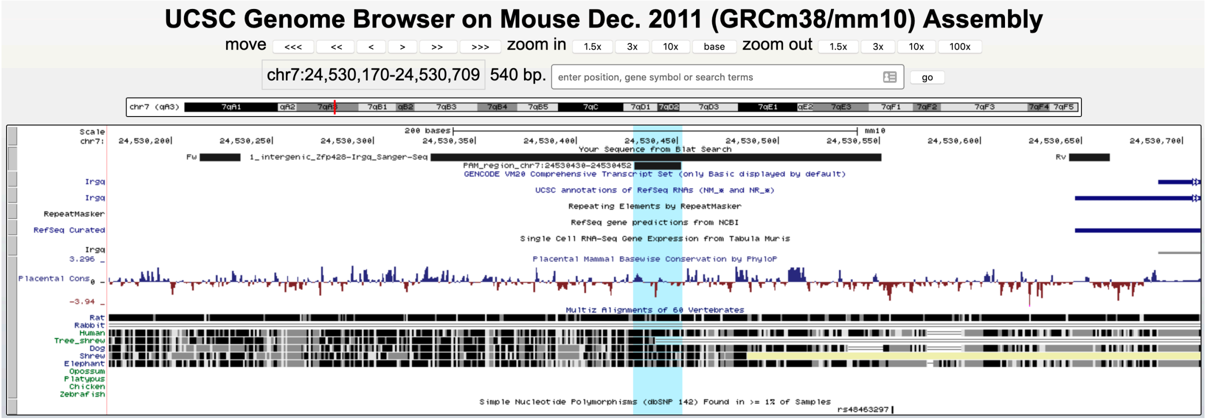

PAM region

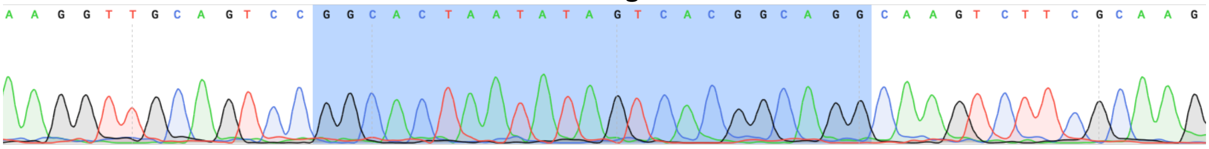

Genomic chr7 (reverse strand):

```
gaaagttttg gggcggtgcc ccaccaagac cagcaaccaa tacgcctgcg   24530602
cagtacgtgc agggtttgta cggcaaaggg ggacaggaag cgatgagccc   24530552
ACGCAGTAAG CTTAGCGTTG TACGGAGCAC ACAACCAACG TCATCATCAC   24530502
GCACCCCTGT ACAGAAAAGG ACCTCTCCAC TTGCCCTTGC GAAGACTTGC   24530452
CTGCCGTGAC TATATTAGTG CCGGACTGCA ACCTTGAACC TGACTCGGGG   24530402
ACAATGGTGG TGCAACAGAT CACTGGAGTC TGGAGACTAG GAAAAACTAT   24530352
CCCGCTCTCG TCCGGTGGCA GAGctgtggc gaccaactgt caggcgcagc   24530302
taagggacag tcttcgggga ggaaagcaag aggcgtccgt gaccatcaag   24530252
aactaagagc agaaggcagt gca
```

cDNA 1_intergenic_Zfp428-Irgq_Sanger-Seq

```
ACGCAGTAAG CTTAGCGTTG TACGGAGCAC ACAACCAACG TCATCATCAC    50
GCACCCCTGT ACAGAAAAGG ACCTCTCCAC TTGCCCTTGC GAAGACTTGC   100
CTGCCGTGAC TATATTAGTG CCGGACTGCA ACCTTGAACC TGACTCGGGG   150
ACAATGGTGG TGCAACAGAT CACTGGAGTC TGGAGACTAG GAAAAACTAT   200
CCCGCTCTCG TCCGGTGGCA GAG
```

Sanger Seq

```
>#2_5prime_intergenic_Gm12705-Dock7_Sanger-Seq
GCCTCTTCTCTGTGAATGGAGATATCAGATGGAACTAGTCTCAAGACAGAAAACAGACTCTGTTGTG
GAAAGCAGCCTCAAACGTGAGTCAGGCTAGCCTTGGAAGTTACAAAATCAAGGCAGGGAAATGAA
GCCTGTGCTGTTGCAGGAGACGTTTAGGTAGACATGGCAGTGATGAGTGATTGCGAGTGGAAGTTG
AGGAAGAGTGGGGCCATGCGCCTTTCC
>Fw
CACCTGAATCCTCACTTCCATCCA
>Rv
AAGGCATGCCATTAAAGGACACA
>PAM_region_chr4:99,119,320-99,119,342
CCTGCCTTGATTTTGTAACTTCC
```

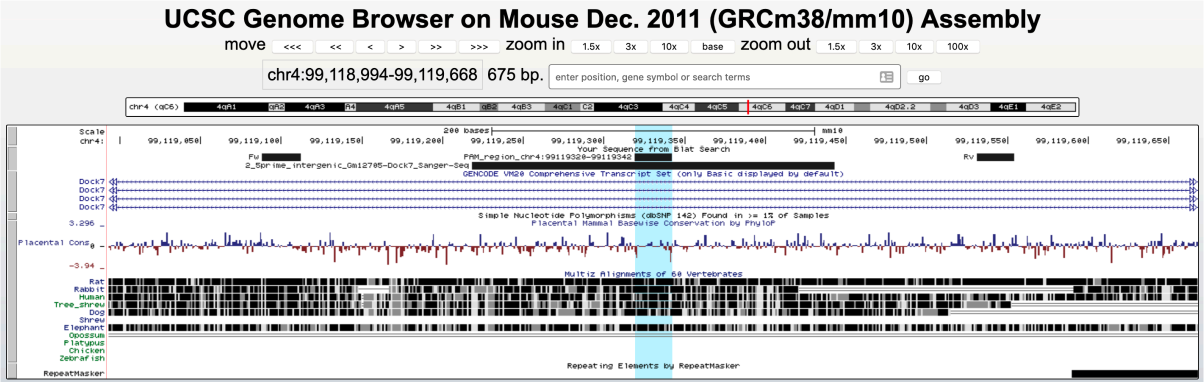

PAM region

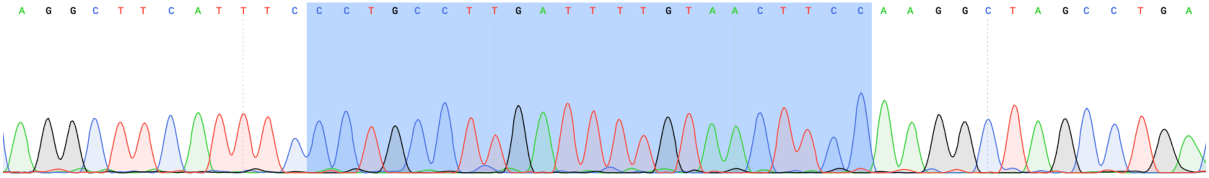

Genomic chr4 (reverse strand):

```
ttaaaggaca cattttgtac aattctttta aatagatctt agagtttata  99119494
tctcataaag taagtacacc cttgagggta aggtatgaaa tatagatagt  99119444
GCCTCTTCTC TGTGAATGGA GATATCAGAT GGAACTAGTC TCAAGACAGA  99119394
AAACAGACTC TGTTGTGGAA AGCAGCCTCA AACGTGAGTC AGGCTAGCCT  99119344
TGGAAGTTAC AAAATCAAGG CAGGGAAATG AAGCCTGTGC TGTTGCAGGA  99119294
GACGTTTAGG TAGACATGGC AGTGATGAGT GATTGCGAGT GGAAGTTGAG  99119244
GAAGAGTGGG GCCATGCGCC TTTCCtgcca cccagctttc cagctgtgca  99119194
gccagctgaa tgatagaagg acatctactc atatctagag ctgtgcatag  99119144
gaggaagtgg tttccgaatt gggga
```

cDNA 2_5prime_intergenic_Gm12705-Dock7_Sanger-Seq

```
GCCTCTTCTC TGTGAATGGA GATATCAGAT GGAACTAGTC TCAAGACAGA  50
AAACAGACTC TGTTGTGGAA AGCAGCCTCA AACGTGAGTC AGGCTAGCCT  100
TGGAAGTTAC AAAATCAAGG CAGGGAAATG AAGCCTGTGC TGTTGCAGGA  150
GACGTTTAGG TAGACATGGC AGTGATGAGT GATTGCGAGT GGAAGTTGAG  200
GAAGAGTGGG GCCATGCGCC TTTCC
```

Sanger Seq

>#3_5prime_intron_Pcsk1_Sanger-Seq

ATCTGCCCTAGAATCCATGTTTTAAGCAGGCATCCAAAGCCTTTCAATGCTTGGAGTACTTTAATCAA
TTACTCTTAAAATTTTTTTTCCTACAACTTCATCCAGCTGTGATTATGAAACTGTCCAATAGGCCACTA
AATAGACAAATTCTGGAGAAAGAAATTCATTACATAAATGCCAGTGGCACCATAATTAGTGTGCATT
AAATACATCTAAGGAAGTTTG

>Fw

TACAGTCAAGCACCAGAGTAGGTG

>Rv

TCCCCTAAAGACTTTGTGCAACAC

>PAM_region_chr13:75,119,264-75,119,286

CCAGCTGTGATTATGAAACTGTC

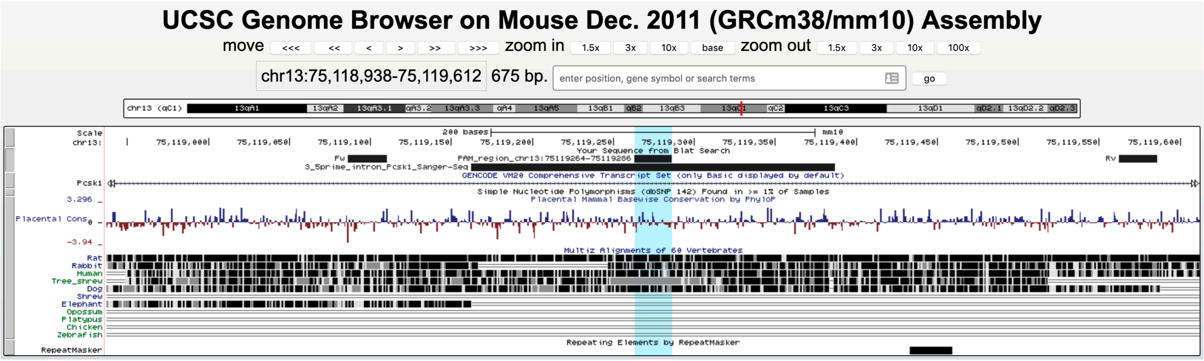

PAM region

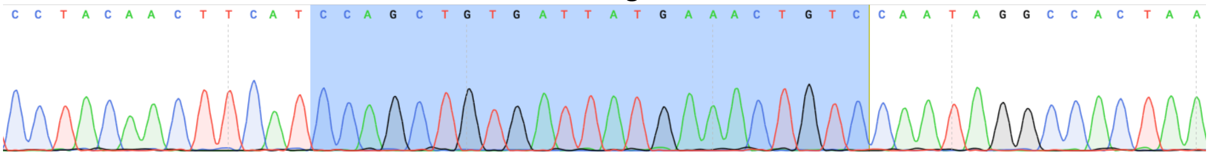

Genomic chr13 :

```
cttccttagt cagttactct tgaatacagt caagcaccag agtaggtgat   75119112
tttccatgta cgttcccaaa tcccattgct aaggactcag attctgaagt   75119162
ATCTGCCCTA GAATCCATGT TTTAAGCAGG CATCCAAAGC CTTTCAATGC   75119212
TTGGAGTACT TTAATCAATT ACTCTTAAAA TTTTTTTTCC TACAACTTCA   75119262
TCCAGCTGTG ATTATGAAAC TGTCCAATAG GCCACTAAAT AGACAAATTC   75119312
TGGAGAAAGA AATTCATTAC ATAAATGCCA GTGGCACCAT AATTAGTGTG   75119362
CATTAAATAC ATCTAAGGAA GTTTGcacac gatcacctct ctcttcagag   75119412
gcaagatcag aagaatcagg gtaaatttta ataataataa aaaaaatctc   75119462
aagtgaacca ttggatacaa tcaca
```

cDNA 3_5prime_intron_Pcsk1_Sanger-Seq

```
ATCTGCCCTA GAATCCATGT TTTAAGCAGG CATCCAAAGC CTTTCAATGC   50
TTGGAGTACT TTAATCAATT ACTCTTAAAA TTTTTTTTCC TACAACTTCA   100
TCCAGCTGTG ATTATGAAAC TGTCCAATAG GCCACTAAAT AGACAAATTC   150
TGGAGAAAGA AATTCATTAC ATAAATGCCA GTGGCACCAT AATTAGTGTG   200
CATTAAATAC ATCTAAGGAA GTTTG
```

Sanger Seq

>#4_5prime_exon_Snx1_Sanger-Seq

ATTCATTCATCTTGATGGTCATTTTGCTGACTGCATCTGTAGCTTTGTTGAACATCTTAAGGAGACCA
GCGCCACTCAAAGCCTGCGTGCCCACAGCTCGAGGCAGCTACAAGATCAAGGCAGGATACATGTTA
TCTGCTGCCCTTTAGGTTCACAAGAATTAGTATCCACCATGGCATGCCCAAAACTTGTCAACAAGTCA
CTTGACTCTAGACTGCTGCATTC

>Fw

GTGCAGGGAGCTGCCTTTCT

>Rv

TGGGCCTGACCACAGAGCAC

>PAM_region_chr9:66,094,731-66,094,753

GGCAGCTACAAGATCAAGGCAGG

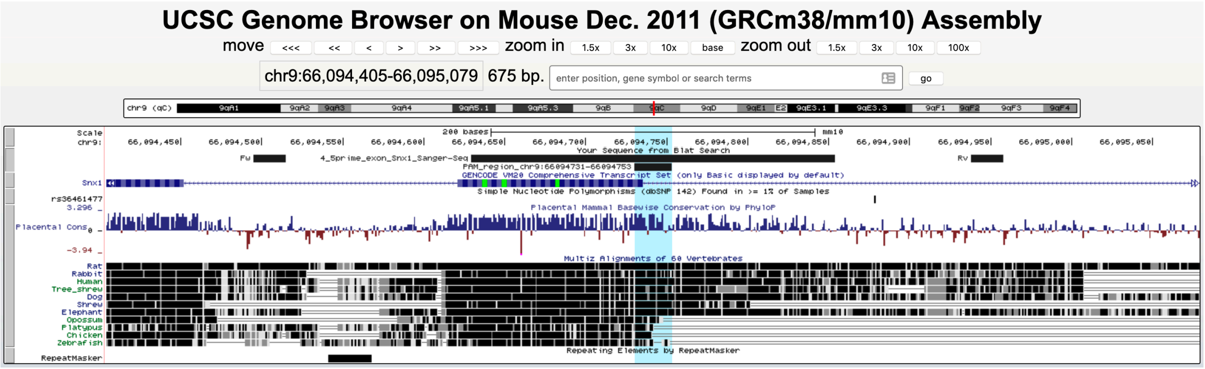

PAM region

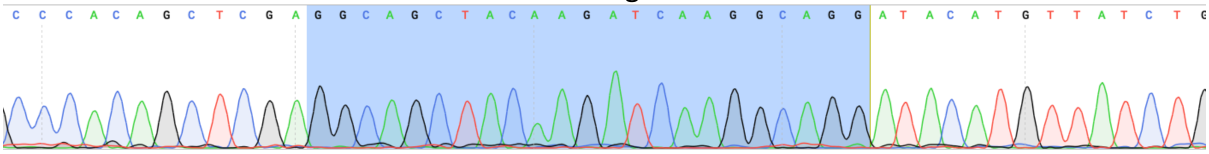

Genomic chr9 :

```
caagggctat tttttgtttg tttgtttgtt tgtttgtttt attcgaaaag  66094579
aaagtaatct cagatatttc aggacttctc agggctactc acaatgtctg  66094629
ATTCATTCAT CTTGATGGTC ATTTTGCTGA CTGCATCTGT AGCTTTGTTG  66094679
AACATCTTAA GGAGACCAGC GCCACTCAAA GCCTGCGTGC CCACAGCTCG  66094729
AGGCAGCTAC AAGATCAAGG CAGGATACAT GTTATCTGCT GCCCTTTAGG  66094779
TTCACAAGAA TTAGTATCCA CCATGGCATG CCCAAAACTT GTCAACAAGT  66094829
CACTTGACTC TAGACTGCTG CATTCgaagg cacttacagg aatctaagat  66094879
taggaaatga gaagcaaacc caaaacacca catactattc acaaaaggaa  66094929
gcacagagag tgctctgtgg tcagg
```

cDNA 4_5prime_exon_Snx1_Sanger-Seq

```
ATTCATTCAT CTTGATGGTC ATTTTGCTGA CTGCATCTGT AGCTTTGTTG   50
AACATCTTAA GGAGACCAGC GCCACTCAAA GCCTGCGTGC CCACAGCTCG  100
AGGCAGCTAC AAGATCAAGG CAGGATACAT GTTATCTGCT GCCCTTTAGG  150
TTCACAAGAA TTAGTATCCA CCATGGCATG CCCAAAACTT GTCAACAAGT  200
CACTTGACTC TAGACTGCTG CATTC
```

Sanger Seq

>#5_5prime_intergenic_C430042M11Rik-Gm23905_Sanger-Seq
GGCTGTCATTATTTTGAGGAAGATCTCTTGAAGCTCAGGCTGGCTTCAGATTAGTTACATAATCAAG
GCTGGCCTTAAGGGCCTGCTTCTCTTACCTTCATCATCCGAGTACTGGAATTATGGGCAAAGCATACA
ACTGTGGCCTGGCTTATATGATAGTTAAAAATTATTAGAATATAGTAGTTGTATCTAATAGTGGGCTC
CATTGCAATATTTCATACA
>Fw
TGGCCCTTATGTGCAGCTCCC
>Rv
AGGGGGATGAGATGGTAGTGGGC
>PAM_region_chr17:83,894,740-83,894,762
ATTAGTTACATAATCAAGGCTGG

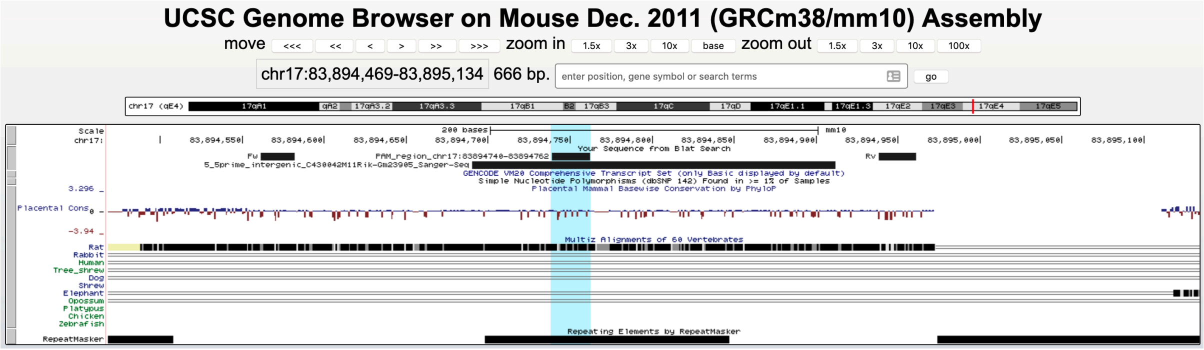

PAM region

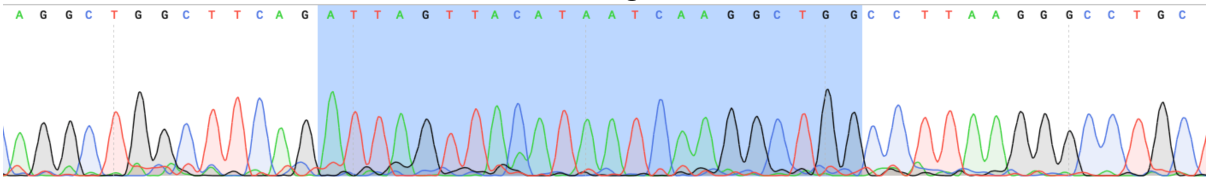

Genomic chr17 :

```
cacctcttct gcccсttcgt ctgccgaagt gacctctagc ctacacttgg  83894640
gctttctcaa tgtccgtgtt tgttcttttc taaagctaca tgcaagtttt  83894690
GGCTGTCATT ATTTTGAGGA AGATCTCTTG AAGCTCAGGC TGGCTTCAGA  83894740
TTAGTTACAT AATCAAGGCT GGCCTTAAGG GCCTGCTTCT CTTACCTTCA  83894790
TCATCCGAGT ACTGGAATTA TGGGCAAAGC ATACAACTGT GGCCTGGCTT  83894840
ATATGATAGT TAAAAATTAT TAGAATATAG TAGTTGTATC TAATAGTGGG  83894890
CTCCATTGCA ATATTTCATA CAtacataat acattttgat catactcagc  83894940
ccactaccat ctcatccccc tccttatgtg tacattttta aaattcattt  83894990
tatttattta tacatgagtg tt
```

cDNA 5_5prime_intergenic_C430042M11Rik-Gm23905_Sanger-Seq

```
GGCTGTCATT ATTTTGAGGA AGATCTCTTG AAGCTCAGGC TGGCTTCAGA  50
TTAGTTACAT AATCAAGGCT GGCCTTAAGG GCCTGCTTCT CTTACCTTCA  100
TCATCCGAGT ACTGGAATTA TGGGCAAAGC ATACAACTGT GGCCTGGCTT  150
ATATGATAGT TAAAAATTAT TAGAATATAG TAGTTGTATC TAATAGTGGG  200
CTCCATTGCA ATATTTCATA CA
```
