# Supplementary material for: A MAFG-lncRNA axis links systemic nutrient abundance to hepatic glucose metabolism
Source: Nat Commun. 2020 Jan 31;11:644. doi: 10.1038/s41467-020-14323-y (PMC6994702; doi:10.1038/s41467-020-14323-y)
Supplement: Supplementary file 17 — Reporting Summary [file 41467_2020_14323_MOESM17_ESM.pdf]

natureresearch

Corresponding author(s): Jan-Wilhelm Kornfeld

Last updated by author(s): 15.12.2019

Reporting Summary

Nature Research wishes to improve the reproducibility of the work that we publish. This form provides structure for consistency and transparency in reporting. For further information on Nature Research policies, see Authors & Referees and the Editorial Policy Checklist .

Statistics

For all statistical analyses, confirm that the following items are present in the figure legend, table legend, main text, or Methods section.

n/a	Confirmed	
[ ]	[x]	The exact sample size (*n*) for each experimental group/condition, given as a discrete number and unit of measurement
[x]	[ ]	A statement on whether measurements were taken from distinct samples or whether the same sample was measured repeatedly
[ ]	[x]	The statistical test(s) used AND whether they are one- or two-sided<br>*Only common tests should be described solely by name; describe more complex techniques in the Methods section.*
[x]	[ ]	A description of all covariates tested
[x]	[ ]	A description of any assumptions or corrections, such as tests of normality and adjustment for multiple comparisons
[ ]	[x]	A full description of the statistical parameters including central tendency (e.g. means) or other basic estimates (e.g. regression coefficient) AND variation (e.g. standard deviation) or associated estimates of uncertainty (e.g. confidence intervals)
[x]	[ ]	For null hypothesis testing, the test statistic (e.g. *F*, *t*, *r*) with confidence intervals, effect sizes, degrees of freedom and *P* value noted<br>*Give P values as exact values whenever suitable.*
[x]	[ ]	For Bayesian analysis, information on the choice of priors and Markov chain Monte Carlo settings
[x]	[ ]	For hierarchical and complex designs, identification of the appropriate level for tests and full reporting of outcomes
[x]	[ ]	Estimates of effect sizes (e.g. Cohen's *d*, Pearson's *r*), indicating how they were calculated

Our web collection on statistics for biologists contains articles on many of the points above.

Software and code

Policy information about availability of computer code

Data collection	Library preparation and sequencing was performed at:<br>(1) Max-Planck Genome-Centre (MP-GC, HFD versus CD cohort)<br>(2) the Cologne Center for Genomics (CCG), Germany (others).<br><br>Following quality checks, 1ug of total liver RNA of each sample was:<br>(1) depleted for rRNA using NEBNext® rRNA depletion Kit (human/mouse/rat). Library preparation was performed with NEBNext Ultra™ Directional RNA Library Prep Kit for Illumina (New England Biolabs).<br>(2) depleted for cytoplasmic and mitochondrial rRNA with Ribo-Zero Gold (LNA Cohort) or Ribo-Zero and strand-specific library preparation performed using TruSeq RNA Gold Kit from Illumina.<br><br>All libraries were sequenced on:<br>(1) HiSeq2500 in 2 x 100bp PE<br>(2) HiSeq 4000 instruments in 2 x 75 PE sequencing mode.
Data analysis	Raw data from Illumina Short Read RNA-Sequencing of mouse liver were processed utilizing the GRCm38 assembly of the mouse genome as gene sets from Ensembl release 90. Biotype and gene features were added manually using Ensembl Biomart. The pipeline consists of six steps:<br><br>(1) barcode and adapter removal using flexbar Version 3.4.0 62<br>(2) computational rRNA depletion by filtering reads that map to known rRNAs in mice using Bowtie2 Version 2.2.963<br>(3i) alignment of non-rRNA reads to the mm10 reference genome using STAR Version 2.6.0c64<br>(4) transcript assembly using cufflinks followed by (5) cuffmerge.<br>(6) cuffdiff performs DGE analysis between experimental conditions via Cufflinks suite Version 2.2.165.

Individual bash scripts were compiled into a Python script. This Python script is available upon request.

For manuscripts utilizing custom algorithms or software that are central to the research but not yet described in published literature, software must be made available to editors/reviewers. We strongly encourage code deposition in a community repository (e.g. GitHub). See the Nature Research guidelines for submitting code & software for further information.

Data

Policy information about availability of data

All manuscripts must include a data availability statement. This statement should provide the following information, where applicable:

- Accession codes, unique identifiers, or web links for publicly available datasets
- A list of figures that have associated raw data
- A description of any restrictions on data availability

Raw data from RNA-Seq are available under GEO ID: GSE121346.
Primary source data from Figures 1-6 and Supplementary Fig 1-5 are available with the manuscript.
Other datasets generated during and/or analysed during the study are available from the corresponding author on reasonable request.

Field-specific reporting

Please select the one below that is the best fit for your research. If you are not sure, read the appropriate sections before making your selection.

[x] Life sciences [ ] Behavioural & social sciences [ ] Ecological, evolutionary & environmental sciences

For a reference copy of the document with all sections, see nature.com/documents/nr-reporting-summary-flat.pdf

Life sciences study design

All studies must disclose on these points even when the disclosure is negative.

Sample size	Sample sizes of mouse cohorts are based on longstanding experience in mouse phenotyping in the corresponding author's group and fall between 6-12 animals per genotype and diet.<br><br>For bulk RNA-Sequencing expression profiling, samples sizes of 3-4 allow robust detection of differential gene expression in complex tissues like liver.<br><br>For primary hepatocyte experiments, we conducted experiments in technical triplicates, which were each performed in three biological replicates (individual mouse liver perfusions).
Data exclusions	No primary data (i.e. outliers) were excluded from the study for statistical reasons.
Replication	Mouse cohorts were not experimentally repeated due to animal welfare reasons and the 3R prinpicles, but each cohort was performed in sufficient statistical power.<br><br>RNA interference experiments in primary hepatocytes and qPCR analyses derive therefrom were performed in technical triplicates, which were each performed in three biological replicates (individual mouse liver perfusions) and all data are shown in the figures.<br><br>RNA interference experiments in primary hepatocytes and immunoblot analyses derived therefrom were performed in technical triplicates, which were each performed in three biological replicates (individual mouse liver perfusions) and all data are shown in the figures.
Randomization	Randomisation of body weight (BW of C57BL/6 experimental mice was performed before exposing mice to experimental diets (Control diet, high-fat diet).
Blinding	Investigators were not blinded to diet information, as body weight gains due to HFD are obvious during the study.<br>During LNA / AAV experiments, investigators were aware of the reagent's nature.<br>During GTT / ITT / PTT, investigators were blinded to the genotype.

Reporting for specific materials, systems and methods

We require information from authors about some types of materials, experimental systems and methods used in many studies. Here, indicate whether each material, system or method listed is relevant to your study. If you are not sure if a list item applies to your research, read the appropriate section before selecting a response.

Materials & experimental systems

n/a	Involved in the study	
☐	☒	Antibodies
☒	☐	Eukaryotic cell lines
☒	☐	Palaeontology
☐	☒	Animals and other organisms
☐	☒	Human research participants
☒	☐	Clinical data

Methods

n/a	Involved in the study	
☒	☐	ChIP-seq
☒	☐	Flow cytometry
☒	☐	MRI-based neuroimaging

Antibodies

Antibodies used

anti-phospho AKTS473 (Cell Signaling, Catalog No. 9271). Dilution 1:1,000
anti-total AKT (Cell Signaling, Catalog No. 4685). Dilution 1:1,000
anti-phospho mTOR S2448 (Cell Signaling, Catalog No. 2971). Dilution 1:1,000
anti-total mTOR (Cell Signaling, Catalog No. 2972). Dilution 1:1,000
anti-phospho 4E-BP1 T37/46 (Cell Signaling, Catalog No. 2855). Dilution 1:1,000
anti-total 4E-BP1 (Cell Signaling, Catalog No. 9452). Dilution 1:1,000
anti-phospho GSK3B S9 (Cell Signaling, Catalog No. 9323). Dilution 1:1,000
anti-total GSK3B (Cell Signaling, Catalog No. 9315). Dilution 1:1,000
anti-NRF1/NFE2L1 (Cell Signaling, Catalog No. 8052). Dilution 1:1,000
anti-MAFG (GeneTex, GTX 114541). Dilution 1:1,000
anti-CLNX (Calbiochem, Catalog No. 208-880). Dilution 1:5,000

Validation

All Cell Signaling antidbodies are standard reagents on the field. The numbers of citations for the individual Catalog numbers on www.citeab.com are (in brackets):
9271 (3872)
4685 (673)
2971 (886)
2972 (751)
2855 (654)
9452 (466)
9323 (268)
9315 (614)

The anti-MAFG antibody was successfully used by the corresponding authors in 'Aiguar de Vallim et al Cell Metab 2015).
Its specificity for MAFG (but not MAFF and MAFK) has been confirmed using lysates from hepatocytes transcfected against Maff, Mafg and Mafk (data available upon reasonable request).

The anti-Calnexin antibody has not been cited before, but was successfully used in other studies by the corresponding author's group (Oliverio Nat Cell Biol 2016, Schmidt Nat Commun 2018).

Animals and other organisms

Policy information about studies involving animals; ARRIVE guidelines recommended for reporting animal research

Laboratory animals

Housing:
- All animals were maintained on a C57BL/6N background, housed in groups of 3-5 animals per cage at 22–24 °C on a constant 12 h light / dark cycle in a SPF controlled facility with regular testing for pathogens.
- Generation of lincIRS2Δ/Δ mice using CRISPR/Cas9 genome engineering is described in Methods section of the paper.
- BKS.Cg-Dock7m+/+;LepRdb/J (db/db) mice and BKS.Cg-Dock7m+/+ (misty/misty) control mice were purchased from Jackson Laboratory or Janvier Labs.

Experimental diet feeding:
- Upon weaning, mice were fed standard rodent chow (Teklad Global Rodent T.2018.R12; Harlan). For experiments involving controlled feeding paradigms, animals were allowed ad libitum access to control diet (CD, D12450B* mod LS; Sniff) containing 62 kJ% carbohydrates, 27 kJ% protein and 11 kJ% fat and drinking water.
- Diet-induced obesity (DIO) was achieved by feeding a high-fat diet (HFD, D12492 (I) mod; Sniff) containing 22 kJ% carbohydrates, 24k J% protein and 54 kJ% fat from starting at 6-7-weeks-of-age. Experimental mice were exposed to specific diets for 10-12 weeks and 17-18-weeks-old at sacrifice unless described below:
- Male, 36-weeks-old C57BL/6N mice exposed to CD (n=3) or HFD (n=3) feeding for 30 weeks, starting at 6-weeks-of-age
- Male, 6-months-old LIRKO (Albumin-Cre+/−, IRflox/flox) and littermate floxed control (Albumin-Cre−/−, IRflox/flox) animals were described in Okada Proc Nat Acad Sci 2007.

	LNA / ASO administration and MAFG AAV8 gain-of-function:<br>- Gender, age and duration of LNA / ASO / AAV8 treatment are provided in the Methods section of the paper.<br><br>Additional information about laboratory animals are available upon reasonable request.
Wild animals	The study did not involve wild animals.
Field-collected samples	The study did not include samples collected in the field.
Ethics oversight	Care of animals was within institutional and animal-care committee guidelines approved by local (Bezirksregierung Köln) or regional (Tierschutzkommission accession no. §15 TSchG of the Landesamt for Natur, Umwelt und Verbraucherschutz (LANUV) North-Rhine Westphalia, Germany, internal reference no. Az-84-02.04-2016.A460) authorities.

Note that full information on the approval of the study protocol must also be provided in the manuscript.

Human research participants

Policy information about studies involving human research participants

Population characteristics	Lean and non-diabetic (n=4) were age 60.75 ± 9 and body mass index (BMI) 23.52 ± 1.36.<br>Obese and non-diabetic (n=4) were age 38.5± 13.42 and BMI 51 ± 2.98.<br>Overweight and diabetic (n=2) age 75.5 ± 0.7, BMI 26 ± 1.41.<br>Obese and diabetic (n=2) were age 44 ± 2.8, BMI 48.25 ± 20.85.<br><br>Additional clinical characterisation of the study cohort is provided in Supplementary Table 4 of (Eissing Nat Commun 2013).
Recruitment	Information on ethical compliance, ethics committee and informed patient consent is described elsewhere (Eissing Nat Commun 2013).
Ethics oversight	Information on ethical compliance, ethics committee and informed patient consent is described elsewhere (Eissing Nat Commun 2013).

Note that full information on the approval of the study protocol must also be provided in the manuscript.
